# Multimodal Multiorgan‐on‐a‐Chip Platform for Probing Liver‐Tumor Interactions and Advancing Prodrug Screening

**DOI:** 10.1002/advs.202600040

**Published:** 2026-04-07

**Authors:** Dan Wang, Yisong Huang, Weijian Zhao, Tian Chen, Siyu Bai, Denghui Guo, Mengying Wang, Yaoyao Zhao, Liang Zhao, Guangsheng Guo, Xiayan Wang

**Affiliations:** ^1^ State Key Laboratory of Materials Low‐Carbon Recycling, Beijing Key Laboratory of Cardiopulmonary‐Cerebral Resuscitation Innovation and Translation, Center of Excellence for Environmental Safety and Biological Effects, Department of Chemistry, College of Chemistry and Life Science Beijing University of Technology Beijing China

**Keywords:** liver metabolism, liver‐tumor interaction, multimodal analysis, multiorgan‐on‐a‐chip, prodrug

## Abstract

To advance the development of microphysiological systems with enhanced metabolic fidelity, there is a critical need to recapitulate native three‐dimensional multicellular architecture, enabling non‐invasive, multi‐parameter measurements. Here, we present a multimodal multi‐OoC platform designed to investigate liver‐tumor interactions and streamline prodrug screening. The platform features a sophisticated microfluidic circuit with pneumatically actuated valves for dynamic perfusion and strategically configured microwell arrays that support the formation and maturation of 3D hepatic constructs. By synergistically incorporating real‐time electrochemical sensing with automated solid‐phase microextraction coupled to mass spectrometry (SPME‐LC‐MS) for pharmaceutical kinetic analysis, the platform allows nonintrusive, longitudinal monitoring of prodrug metabolism. We further implemented an impedance‐based immunosensor for real‐time assessment of drug‐induced hepatotoxicity, quantifying secreted albumin across clinically relevant concentrations (1–80 µM). As proof of concept, we evaluated the metabolic activation and subsequent antineoplastic efficacy of two prodrugs, capecitabine and tamoxifen, highlighting the system's ability to elucidate hepatic metabolic activation pathways and resultant antineoplastic efficacy. By unifying 3D tissue models with complementary real‐time analytical modalities, this work provides a versatile and transformative approach for in vitro drug evaluation and mechanistic organ‐organ interaction studies.

L. Z. conceived the study. D. W. performed the experiments, analyzed the data, and wrote the manuscript. L. Zhao. wrote and revised the manuscript. X. W., and G. G. helped on revising the manuscript. D. G., M. W., and Y. Z. helped to perform the mass spectrometry testing of the parent drug and its metabolites. T. C. assisted in completing all the work related to the proteomics section. Y. H. and S. B. assisted in the fabrication and characterization of the chip. L. Z. and X. W. supervised the research project. All authors read and approved the final manuscript.

## Graphical abstract

This work introduces a multimodal multi‐OoC platform that overcomes current limitations in liver‐tumor interaction studies and prodrug screening. By integrating dynamic microfluidic circuits, electrochemical sensing, and mass analysis, this platform enables non‐invasive, longitudinal monitoring of drug metabolism and hepatotoxicity, offering a transformative approach for in vitro drug evaluation and mechanistic studies.

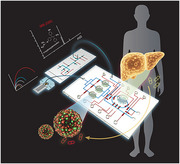

## 1 Introduction

Heavy financial input, protracted timelines, and significant attrition rates characterize the development of new drugs [1]. Despite substantial investment, many promising drug candidates fail during clinical trials, primarily due to inadequate initial toxicity screening that leads to unexpected adverse reactions across multiple organs [2, 3]. The off‐target hepatotoxicity represents a particularly significant challenge, accounting for approximately 40% of drug failures during development [4]. The accurate prediction of drug‐induced hepatotoxicity is therefore a critical step in reducing costs and attrition rates, especially during the early phases of pharmaceutical development [5]. Thus, the comprehensive evaluation of drug candidates is a prerequisite during the non‐clinical development stages. This challenge becomes even more pronounced when considering prodrug candidates, which present unique screening complexities.

Prodrugs are pharmacologically inactive or partially active compounds that undergo in vivo conversion to release the active [6, 7]. The evaluation of prodrugs introduces additional layers of complexity to toxicity screening. Traditional animal‐based drug testing approaches show significant limitations in prodrug assessment due to inherent species differences in metabolism and genetic heterogeneity [8]. These biological variations often lead to discrepancies between animal models and human responses, making it difficult to accurately predict prodrug activation, metabolism, and potential hepatotoxicity in human patients [9, 10]. Furthermore, prodrugs require specific enzymatic transformations to convert the inactive parent compound into pharmacologically active metabolite‐processes that can vary substantially between species and even among human populations with different genetic polymorphisms [11]. Conventional in vitro models also fall short in replicating the complex inter‐organ interactions necessary for comprehensive prodrug evaluation, as they typically focus on isolated cell types or single organs [12, 13]. Current approaches often rely on simplified techniques, such as supernatant transfer between discrete tissue compartments, which inadequately recapitulate the dynamic inter‐organ crosstalk required for accurate prediction of human responses and subsequent safety evaluation [14, 15]. Despite their potential, the intrinsic complexity of prodrugs, characterized by their dependency on enzymatic bioactivation, presents significant challenges for comprehensive in vitro assessment [16, 17]. The biotransformation of prodrugs often involves multiple organ systems working in concert, with metabolites potentially causing toxicity in tissues distant from the site of activation [18]. This systemic nature of prodrug metabolism and toxicity highlights the critical need for advanced testing platforms that can more faithfully recapitulate the orchestrally interconnected organs of the human body.

Multi‐organ‐on‐a‐chip (multi‐OoC) technologies have emerged as promising alternatives to conventional testing paradigms, facilitating the integration of engineered human tissues with physiologically relevant intercellular communication networks [19, 20, 21, 22, 23, 24]. The ability to fluidically link multiple miniature 3D microscale organs for interactional studies has established organ‐on‐a‐chip (OoC) systems as widely adopted, powerful in vitro models for drug development and therapeutics screening [25, 26, 27]. Several examples have demonstrated the feasibility of using multi‐OoC methods to assess the safety and efficacy of prodrugs [28, 29]. However, measuring prodrug metabolites and liver status in real time is challenging without an integrated detection module, which hinders their implementation in large‐scale pharmaceutical applications. In terms of effective prodrug screening, several critical physiological features must be faithfully emulated: i) compartmentalized cultivation of human tissues maintaining dynamic homeostatic regulation, ii) three‐dimensional architectural organization of cellular components with enhanced metabolic functionality, and iii) continuous, non‐invasive monitoring capabilities for tissue viability assessment and identification of reactive metabolites.

Despite recent advancements, contemporary multi‐OoC platforms exhibit limitations in their capacity to reconstitute the intricate three‐dimensional hepatic architecture necessary for efficient prodrug bioactivation and subsequent therapeutic assessment [30, 31]. These systems often exhibit insufficient metabolic fidelity, compromised tissue‐tissue interfaces, and inadequate perfusion dynamics, thereby undermining their predictive capabilities [32, 33, 34, 35, 36]. Multiple platforms struggle to accurately mimic the complex three‐dimensional architecture of the liver, with its specialized zonation and multicellular composition [37, 38]. Critically, enhanced metabolic functionality in these platforms has rarely been experimentally verified in the aspect of prodrug activation. Current platforms also demonstrate limited capacity to maintain physiological homeostasis during extended culture periods, a prerequisite for evaluating delayed hepatotoxicity that may manifest only after prolonged exposure [39]. Furthermore, most OoC systems still rely on endpoint assays and lack the capability for real‐time monitoring of cellular responses to prodrug administration and consequent liver injury, including the crucial process of metabolite generation [40, 41, 42]. Although some systems permit the non‐invasive measurement of organ‐specific toxicities through the monitoring of oxygen levels [43, 44], cellular metabolic dysfunction [45], ATP variation [46], and transepithelial electrical resistance [47], the continuous and chemical analysis of metabolite transformation processes via mass spectrometric implementation remains a significant technical challenge, underscoring the urgent necessity for advanced biomimetic multi‐OoC systems engineered explicitly for comprehensive prodrug evaluation.

To address these limitations, we introduce iMULTI‐chip, an integrated multi‐OoC platform engineered for investigating liver‐tumor interactions with comprehensive, multimodal analytical capabilities. Our methodology is motivated by two ongoing challenges associated with the development and implementation of a multi‐OoC system to study the crosstalk between different organs, especially liver‐tumor interactions. First, how to recapitulate a physiological realism in the biological complexity of tissue architecture, hemostasis, and metabolism, which is essential for prodrug screening. Second, creating a highly controllable system with direct incorporation of multiplex non‐disruptive chemical measurement continues to be a critical yet challenging issue. The iMULTI‐chip achieves true multimodal analytical integration that combines physiologically relevant organ‐organ crosstalk with simultaneous, orthogonal biochemical interrogation, representing a paradigm shift from conventional OoC systems that rely solely on endpoint optical readouts or require destructive sample collection for external analysis. The platform integrates three independently addressable microfluidic circuits within a unified architecture: i) a pneumatically actuated dynamic circulatory network establishing enhanced homeostasis and directional liver‐tumor communication through controlled perfusion, with microwell arrays facilitating spontaneous spheroid aggregation, ii) an on‐chip automated solid‐phase extraction (SPE) module directly interfaced with mass spectrometry, representing the first online chemical measurement integrated within OoC prodrug testing, and iii) embedded electrochemical impedance sensors for real‐time monitoring of hepatotoxicity biomarkers (albumin secretion) without sample extraction. To this end, we have conducted proof‐of‐principle experiments including landscaping hepatic spheroid proteomics, assessment of acetaminophen‐induced hepatotoxicity (decreased albumin secretion) across clinically relevant drug concentrations (1–80 µM) without sample extraction, and quantitative detection of capecitabine conversion and tamoxifen metabolism. Such a multimodal analytical framework addresses the critical gap of conventional platforms, which must sacrifice tissue for analysis, precluding temporal correlation between metabolic conversion and downstream therapeutic effects. The iMULTI‐chip system uniquely correlates the prodrug activation in liver spheroids, metabolite accumulation in circulating media, and cytotoxic response in tumor spheroids, all from the same biological replicates over extended time courses. This investigation represents a significant advancement in OoC pharmacological testing methodologies through the integration of physiologically relevant three‐dimensional tissue models, where understanding tissue‐specific metabolism and organ crosstalk remains a critical bottleneck in predicting human responses.

## 2 Results and Discussion

### 2.1 The Design and Principle of iMULTI‐Chip

**FIGURE 1:**
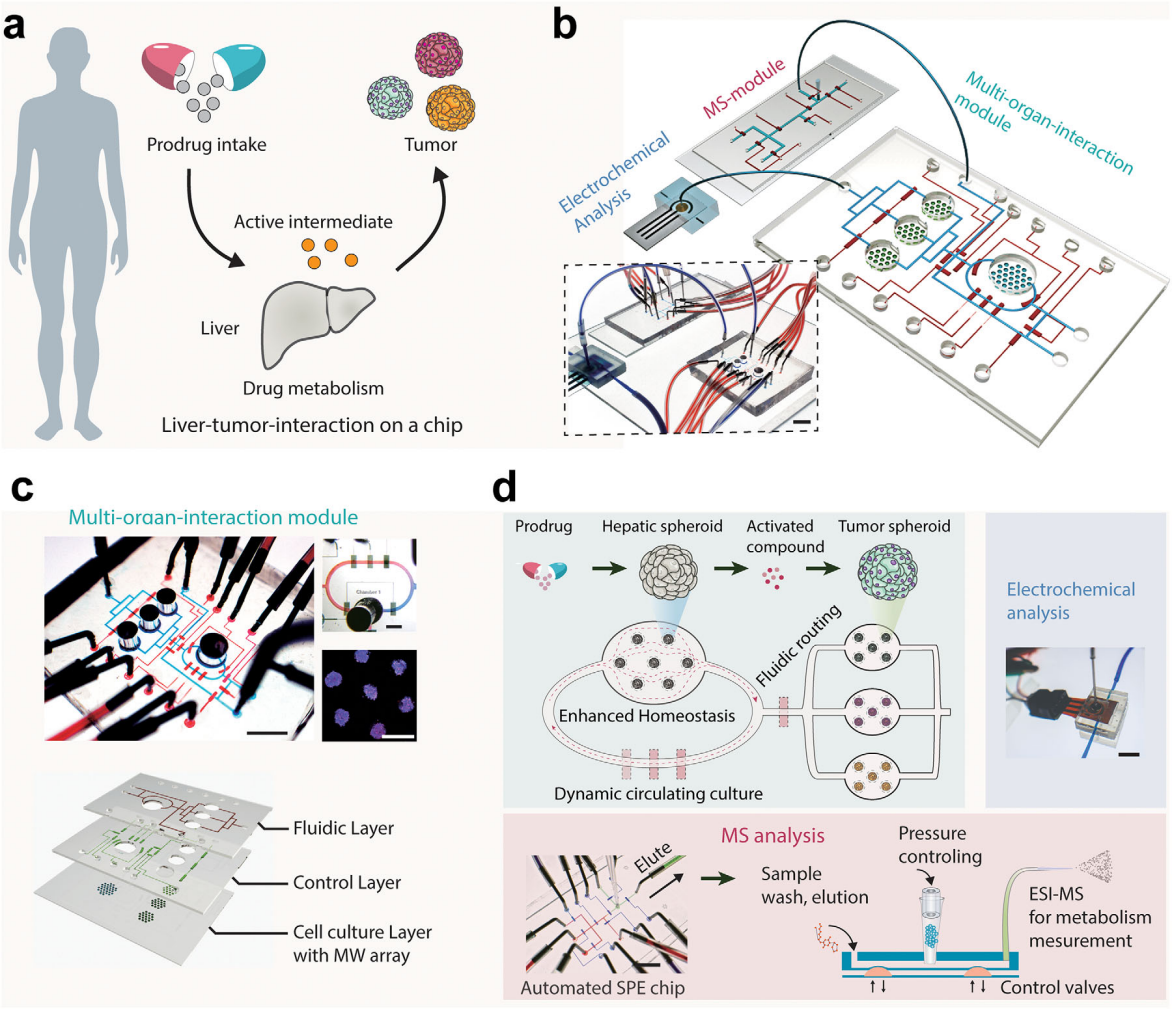
The integrated multi‐OoC with multimodal analysis (iMULTI‐chip) platform for prodrug screening. (a) Schematic depicting the liver‐tumor pharmacokinetic interaction during prodrug intake and metabolism. (b) Integrated microfluidic platform architecture featuring three chips, the electrochemical immunosensing analysis, mass spectrometry, and a multi‐organ‐interaction module with interconnected microchannels. Insert: photograph of the iMULTI‐chip system. (c) Multi‐organ‐interaction module construction: top micrograph showing the microfluidic layout; inset: the microfluidic circulation unit for liver spheroids culture, and immunofluorescence of the 3D hepatic spheroids in microwell arrays (Albumin/DAPI merge, scale bar: 1 mm); bottom: trilayer microfluidic device comprising fluidic, control, and cell culture layers; exploded view showing the trilayer configuration (bottom). (d) Top micrograph showing the multi‐organ microfluidic configuration illustrating the experimental design: administration of prodrugs and hepatotoxic agent to liver spheroids under dynamic circulatory treatment, with subsequent exposure of metabolites to downstream tumor spheroid arrays; inset: the electrochemical sensor module connected to the liver‐tumor Interaction microfluidic chip; bottom: integrated ESI‐MS module (graphic image) with pneumatic flow control for online automated metabolite quantification. The schematic illustration of solid‐phase microextraction (SPME) shows the pneumatic control of the sampling, washing, and elution processes. Scale bar is 5 mm.

The goal of our methodology was to develop a highly functional, perfusion‐driven integrated microfluidic multi‐OoC system comprising a 3D liver and tumor with multimodal analytical technologies. Thus, the iMULTI‐chip platform was designed to allow physiological function and communication with four capabilities: i) on‐chip aggregation and dynamic culture of hepatic cells and tumor cells; ii) an microfluidics chip with highly tunable fluidic control for closed‐loop circulating hepatic spheroid culture and time‐dependent prodrug dosing; iii) an integrated electrochemical sensor device for online analysis of hepatic cell injury in a modular manner; iv) a valve‐based modular microfluidics circuit for online micro solid phase extraction and mass spectrometric analysis. As shown in Figure 1a, the liver‐mediated prodrug transformation and metabolic activity are critical in pharmaceutical medication studies. Our microfluidics devices were fabricated using conventional polydimethylsiloxane (PDMS) soft lithography and molding techniques [48]. The orthogonal intersection of flow and control lines resulted in the formation of several pneumatic, PDMS‐based, push‐up membrane valves. The modular design integrates three functional components: electrochemical analysis, mass spectrometry, and multi‐organ interaction modules, all interconnected through controlled microfluidic channels and tycoon tubing (Figure 1b). This tri‐module approach facilitates comprehensive analysis of the entire pharmacokinetic pathway from prodrug administration to metabolite formation and tumor targeting. The trilayer configuration shown in Figure 1c (fluidic layer, control layer, and cell culture layer) demonstrates sophisticated engineering that separates the liver and tumor niche from the control mechanisms, allowing for the precise regulation of flow rates and fluidic control from biological components while maintaining physiological communication.

Notably, the microwell array design supports 3D spheroid formation, which more accurately recapitulates in vivo cellular organization than traditional 2D cultures. The culture array structure consists of microcavities with a height of 300 micrometers and a diameter of 300 micrometers. The multi‐organ co‐culture chip features a fluid layer with one inlet, two outlets, and a bubble vent; a control layer with 12 control valves; and an array layer containing one hepatocyte spheroid culture chamber and three parallel tumor spheroid culture chambers. The open configuration, combined with a microwell array, allows easy cell seeding and subsequent biological measurements (see details in Figures S1–S4; Videos S1–S3). Moreover, the closed‐loop circulation system mimics vivo blood circulation, recapitulating the homeostasis of liver units and allowing metabolites generated by hepatic spheroids to reach tumor spheroids in physiologically relevant concentrations and time frames (Figure 1c). This is particularly crucial for studying prodrugs, which rely on hepatic metabolism for activation. Immunofluorescence visualization confirms the successful formation of hepatic spheroids with functional albumin production, indicating that liver‐specific functions are preserved within the microenvironment. Figure 1d illustrates the sophisticated on‐chip multimodal platform designed for a dynamic co‐culture circuit, MS analytical module, and electrochemical module. In this configuration, prodrugs are administered to hepatic spheroids, with the resulting metabolites subsequently partitioned to a downstream tumor spheroid array. The integrated human albumin antibody‐modified electrochemical sensors enable seamless monitoring of liver injury biomarkers.

To enable non‐destructive monitoring of hepatocyte spheroid metabolism, we developed an integrated microfluidic chip coupling automated solid‐phase microextraction (SPME) with liquid chromatography mass spectrometry (LC‐MS) tandem detection (Figure 1d). The device employs a three‐layer architecture optimized for automated sample processing (SPME) and MS analysis. The chip comprises: i) a fluidic layer housing the SPE module and sample channels, ii) a pneumatic control layer for valve actuation, and iii) a glass substrate providing structural support and optical transparency. The integrated SPME unit utilizes C18‐functionalized pipette tips as the stationary phase, eliminating traditional packed‐bed limitations. Each extraction tip is interfaced with flexible silicone tubing and controlled via pneumatic microvalves. This configuration enables bidirectional flow control‐pressurized air (tip valve) drives sample elution from the C18 sorbent into the microfluidic channels, while valve deactivation permits sample loading through the chip inlet, pushing from the co‐culture chip. Such an operation can be expanded to the washing step of online SPME as well. At the outlet of the fluidic layer, a capillary tube is connected to the liquid injection vial, followed by liquid chromatography‐mass spectrometry (LC‐MS) analysis, achieving end‐to‐end integration of extraction and mass spectrometric detection. This “sample‐to‐spectrum” integration achieves continuous metabolite monitoring while maintaining spheroid viability. The platform's key innovation lies in its ability to perform sequential, non‐invasive metabolomic profiling, capturing temporal dynamics of drug metabolism and cellular response that are inaccessible to endpoint assays in conventional drug screen systems.

### 2.2 Liver‐on‐a‐Chip With Dynamic Recirculating Flow

**FIGURE 2:**
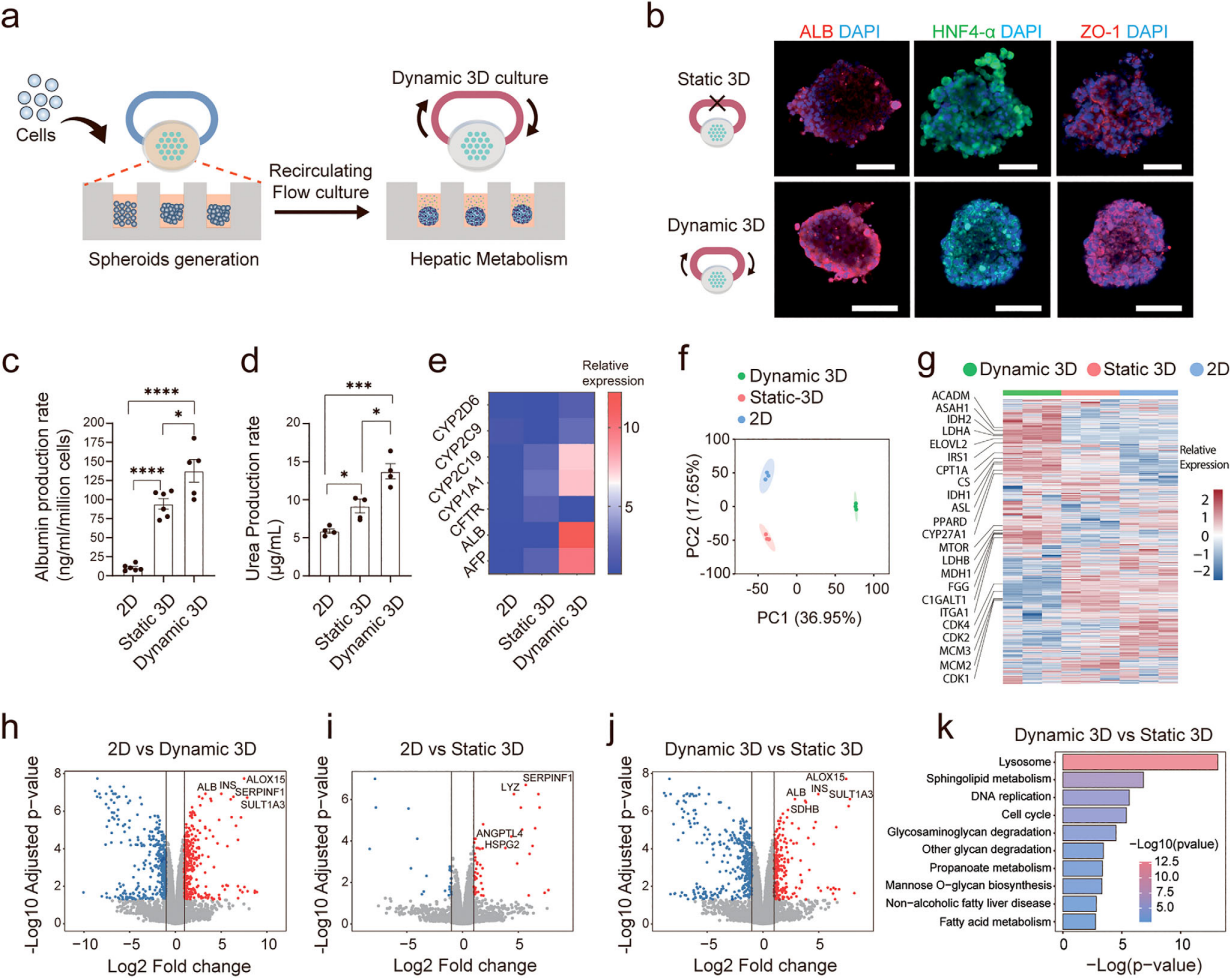
Comparative analysis of hepatocyte functionality in 2D, static 3D, and dynamic 3D culture systems. (a) Schematic workflow illustrates spheroid generation and integration into recirculating flow culture. (b) Immunofluorescence micrographs showing expression of albumin (ALB, red), hepatocyte nuclear factor 4‐alpha (HNF4‐α, green), and zonula occludens‐1 (ZO‐1, red) with nuclear counterstaining (DAPI, blue) in static versus dynamic 3D cultures. Scale bar: 100 µm. (c,d) Quantitative assessment of liver‐specific functions: (c) albumin production (ng/mL/million cells) and (d) urea secretion rates (µg/mL) across culture modalities. (e) Heatmap visualization of hepatic‐specific gene expression profiles, including cytochrome P450 enzyme genes. The gene β‐actin was used for normalization of the qPCR results. (f) Principal component analysis (PCA) demonstrates proteomic clustering of the three culture paradigms. (g) Proteomic heatmap of metabolic gene expression profiles across culture conditions. (h–j) Volcano plots depicting differential gene expression: (h) 2D versus dynamic 3D, (i) 2D versus static 3D, and (j) dynamic 3D versus static 3D cultures. (k) Pathway enrichment analysis highlighting significantly upregulated biological processes in dynamic 3D compared to static 3D culture. Data presented as mean ± SEM, n = 3, P‐values are calculated using one‐way ANOVA with Tukey's post‐hoc test analysis, ^*^ *p* < 0.05, ^***^ *p* < 0.001, ^****^ *p* < 0.0001. Proteins with a fold change > 1.5 or < 0.67 and a *p*‐value < 0.05 were considered significantly differentially expressed.

We strategically developed the liver/tumor‐on‐a‐chip as the organ interaction and prodrug testing module, as the liver plays a pivotal role in metabolic physiology. Unlike the previous 2D monolayer liver [49] or 3D tissue in a hydrogel [50], we prioritized the 3D tissue architecture and dynamic homeostasis to enhance the in vitro cultured liver function, particularly its metabolic capability. To this end, a closed‐loop, recirculating microfluidic format with a microwell array structure has been employed, enabling long‐term, sustainable perfusion dynamic culture (Figure 2a). By alternatively actuating three pneumatic valves with an interval, such a configuration enables peristaltic pumping, allowing the solution in the closed microfluidic channel to be manipulated in a circulation manner (Figure S5, Videos S4 and S5). The HepaRG cell suspension was pipetted into the microwells from the open chamber after the whole device had been primed with culture medium. The multicellular spheroids were generated after 12–24 h of static culture and then treated with dynamic looping culture for an additional 48 h or a more extended period.

We next sought to confirm that our dynamic 3D culture modality allows enhancement of liver function. Immunofluorescence analysis revealed that hepatocytes cultured in the dynamic 3D system exhibited significantly enhanced expression of key hepatic markers, including albumin (ALB), hepatocyte nuclear factor 4‐alpha (HNF4‐α), and tight junction protein zonula occludens‐1 (ZO‐1), compared to their static 3D counterparts (Figure 2b) and the 2D monolayer culture (Figure S6). Quantitative assessment of liver‐specific functions demonstrated that dynamic 3D culture resulted in approximately threefold higher albumin secretion rates (∼60 ng/mL/million cells) compared to static 3D culture (∼20 ng/mL/million cells) and 2D culture (∼15 ng/mL/million cells) (Figure 2c). Similarly, urea production was substantially elevated in dynamic 3D conditions (∼15 µg/mL) relative to static 3D (∼10 µg/mL) and 2D (∼5 µg/mL) cultures (Figure 2d). Moreover, qPCR analysis revealed differential gene expression patterns across culture modalities, with cytochrome P450 enzymes (CYP2D6, CYP2C9, CYP2C19, CYP1A1) showing progressive upregulation from 2D to static 3D to dynamic 3D conditions (Figure 2e). The mouth pipetting with a pulled open capillary was used to precisely pick up a single cell aggregate from the culture chamber, which has been opened up for facilitating spheroid retrieval (Figure S7) [51].

To further elucidate the molecular mechanisms underlying functional enhancement, we performed comprehensive proteomic profiling across different culture conditions. Principal Component Analysis (PCA) revealed that the Dynamic 3D group possessed a distinct proteomic signature, separating significantly from the 2D and Static 3D groups along the first principal component (Figure 2f). Hierarchical clustering and heatmap analysis further illustrated that while 2D cultures remained in a highly proliferative state (indicated by elevated cell cycle proteins such as CDK1 and MCM2), the dynamic 3D environment promoted the expression of mature hepatic markers and metabolic enzymes, including ACADM and CPT1A (Figure 2g).

Volcano plot analysis confirmed a robust up‐regulation of functional proteins in the Dynamic 3D group compared to 2D and Static 3D (Figure 2h–j) counterparts, with significant enrichment of key proteins such as ALB, ALOX15, and SULT1A3. Furthermore, KEGG pathway enrichment analysis of the differentially expressed proteins between the Dynamic 3D and Static 3D groups (Figure 2k) highlighted a significant activation of fatty acid metabolism, sphingolipid metabolism, and lysosomal pathways. These findings collectively demonstrate that the integration of 3D architecture with recirculating flow effectively suppresses the dedifferentiation seen in 2D cultures and drives the hepatic spheroids toward a more mature, metabolically active phenotype that closely mimics the physiological state of the liver in vivo. Venn diagram analysis further confirmed that the dynamic 3D environment induced the highest number of unique differentially expressed proteins (1338), exceeding those in static conditions (Figure S8). Compared to the Static 3D group, the Dynamic 3D group showed significantly superior activation of fatty acid β‐oxidation, oxidative phosphorylation, mitochondrial respiratory chain activities, and lysosomal functions (Figures S9, S10, S12 and S13). Sankey diagrams effectively visualized the massive recruitment of functional proteins into lipid and carbon metabolism pathways (Figure S11). Collectively, we analyzed the proteomic landscape and the results evidently showed that the dynamic recirculating system significantly enhances hepatocyte metabolic maturation, particularly in pathways related to energy metabolism, xenobiotic metabolism, and lipid processing, which are pivotal for prodrug activation in liver‐tumor interaction.

### 2.3 The Electrochemical Analysis of Drug‐Induced Liver Injury

**FIGURE 3:**
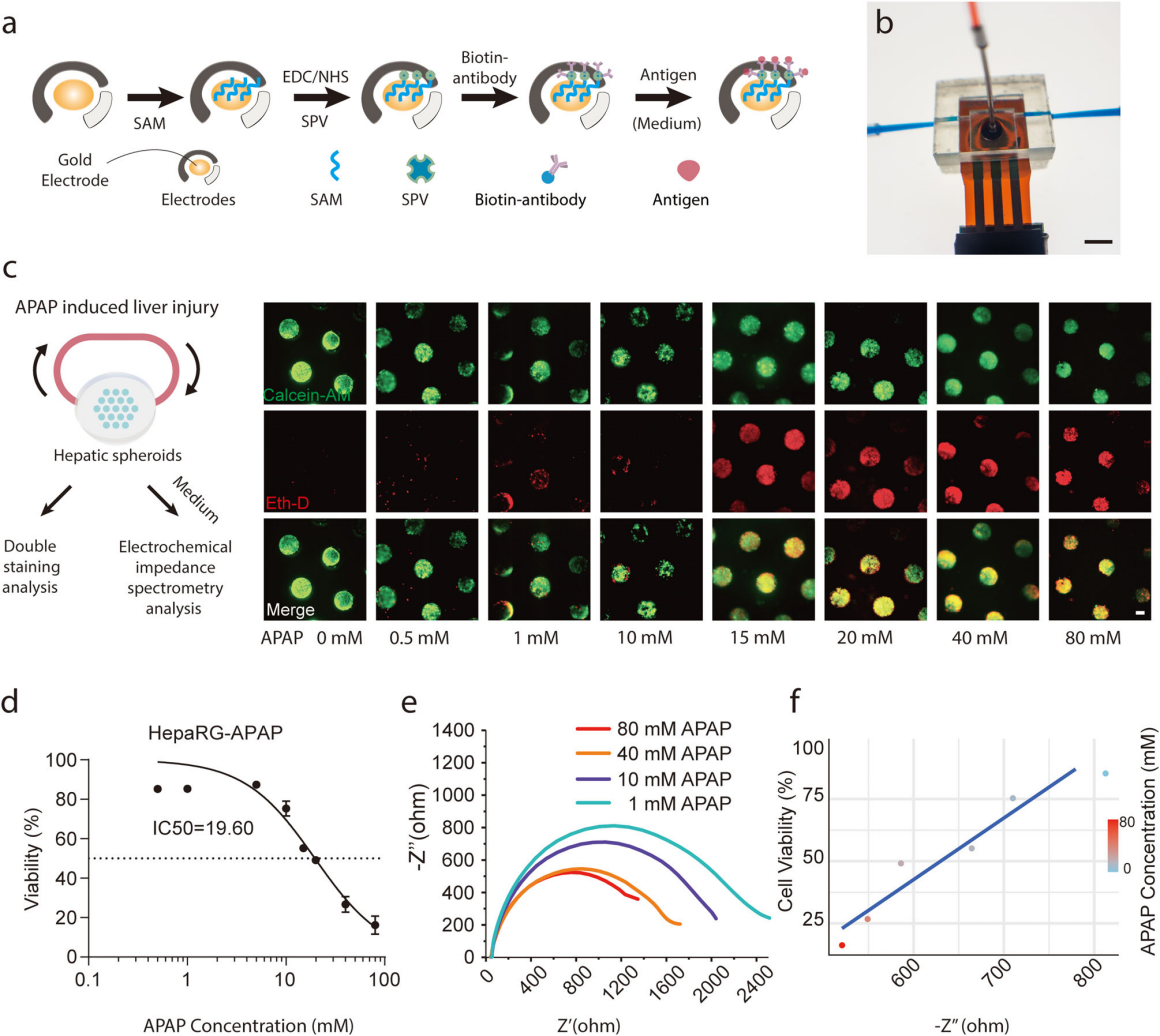
Integration of electrochemical biosensing for quantitative evaluation of drug‐induced hepatotoxicity in 3D hepatic spheroids. (a) Schematic illustration of sequential electrode modification for electrochemical impedance‐based biosensing: gold electrode modification with self‐assembled monolayer (SAM), activation via EDC/NHS chemistry, attachment of signal peptide vector (SPV), immobilization of biotinylated antibody, and subsequent antigen capture from culture medium. (b) Photographic illustration of the electrochemical sensor module connected to the liver‐tumor Interaction microfluidic chip. Scale bar: 5 mm. (c) Schematic depiction of the drug‐induced liver injury (DILI) assay using fluorescence‐based double staining and electrochemical impedance spectrometry (EIS) analysis, with representative fluorescence micrographs showing acetaminophen (APAP) induced hepatic toxicity across a concentration gradient (0–80 mM). Live cells visualized with Calcein‐AM (green, top row), dead cells with Ethidium homodimer‐1 (Eth‐D, red, middle row), and merged images (bottom row). Scale bar: 100 µm. (d) Quantitative concentration‐response analysis of HepaRG cells treated with APAP, disclosing a sigmoidal toxicity profile with a calculated IC_50_ of 19.60 mM (mean ± SEM, n = 3). (e) Nyquist plots of impedance spectroscopy measurements corresponding to varying APAP concentrations (1–80 mM), reflecting concentration‐dependent alterations in hepatic albumin secretion. (f) Linear regression analysis (R^2^ = 0.93) was performed between the imaginary impedance component (‐ Z″) and viability, along with APAP concentration (color gradient from 0 to 80 mM), validating impedance‐based toxicity sensing. Statistical significance was assessed using Pearson correlation analysis, yielding a correlation coefficient of r = 0.961 (*p* < 0.001). Data presented as mean ± SEM, n = 3.

Drug‐induced liver disease, also known as drug‐induced liver injury (DILI), is a significant clinical challenge and a leading cause of acute liver failure [52]. For example, acetaminophen is safe at therapeutic doses but can cause severe liver injury or even acute liver failure when taken in excess [53]. To address this critical issue, we integrated an electrochemical sensing module into our iMULTI‐chip system, enabling real‐time, label‐free monitoring of hepatotoxicity (Figure 3a, b). We chose the secreted albumin as a biomarker of liver health [54, 55, 56]. The sensor assembly consisted of a screen‐printed three‐electrode system with a functionalized gold electrode featuring self‐assembled monolayers (SAMs) and biotin‐albumin antibody conjugates for the specific detection of human albumin in the culture medium. The modified screen‐printed electrodes were inserted into a 3D printed microfluidic device with two inlets and one outlet, as shown in Figure 3b, Figure S14. Electrochemical characterization validated the stepwise electrode modification. Cyclic voltammetry (CV) showed a progressive decrease in peak currents, while EIS Nyquist plots revealed a corresponding increase in charge transfer resistance from the bare electrode to the antibody‐functionalized surface. (Figure S15).

We validated this approach using acetaminophen (APAP), a well‐characterized hepatotoxicant that causes dose‐dependent liver injury. This experiment was conducted as a high‐dose stress validation model. Hepatic spheroids exposed to increasing APAP concentrations (0–80 mM) demonstrated a progressive loss of viability, as visualized by Calcein‐AM/Ethidium‐D staining, with minimal toxicity observed at 0.5–1 mM, moderate injury at 10–15 mM, and extensive cell death at concentrations of ≥40 mM (Figure 3c). Quantitative analysis established a characteristic sigmoidal concentration‐response relationship with an IC_50_ value of 19.60 mM in HepaRG cells (Figure 3d), consistent with previously reported hepatotoxicity thresholds for metabolically competent hepatic models [57]. In addition, we investigated the impact of prodrug capecitabine (CAP) and acetaminophen (APAP) treatment on liver spheroids in our device. We employ proteomic analysis to elucidate the underlying mechanisms of these two drugs. The comparison results of the volcano plot, unsupervised clustering of protein expression, KEGG, and Sankey diagram of KEGG, revealed that APAP and CAP induce distinct proteomic signatures reflecting their different mechanisms of action and hepatic effects (Figures S16–S21). Generally, these results confirm that our liver‐on‐chip platform can effectively distinguish between different hepatotoxic mechanisms. APAP predominantly induces mitochondrial and ER stress pathways characteristic of acute hepatotoxicity, while CAP primarily affects nucleic acid metabolism pathways, consistent with its antimetabolite mechanism of action.

Electrochemical impedance spectroscopy (EIS) revealed distinctive Nyquist plots across APAP concentrations, where higher drug concentrations led to a significant decrease in impedance values, reflecting dose‐dependent hepatotoxicity. (Figure 3e). This inverse relationship between APAP concentration and impedance parameters reflects compromised cellular integrity and reduced albumin secretion, resulting in lower antigen‐antibody reaction following hepatocyte injury. Linear regression analysis demonstrated a strong correlation (R^2^ = 0.93) between the imaginary impedance component (‐Z“) and APAP concentration, with ‐Z” values ranging from approximately 600 to 800 Ω corresponding to viability percentages of 25%–90% (Figure 3f). This robust correlation validates our electrochemical platform as a reliable and quantitative method for non‐invasive monitoring of hepatotoxicity, with impedance values directly corresponding to the functional status of hepatocytes, potentially enabling the longitudinal assessment of drug‐induced injury in complex liver‐tumor interaction models without disrupting the culture environment.

### 2.4 Online Mass Spectrometric Analysis of Prodrug Metabolism

**FIGURE 4:**
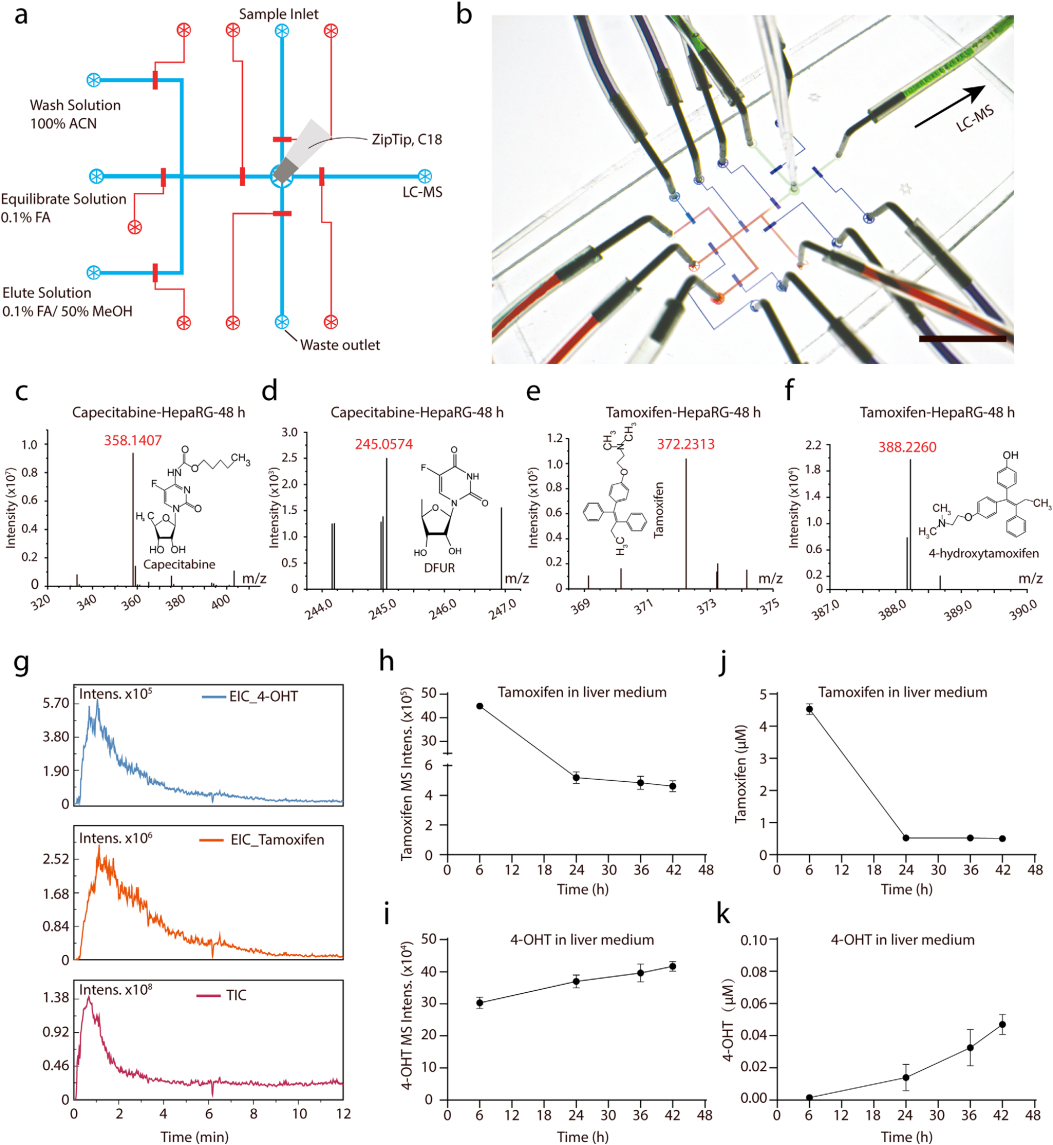
Integration of automated solid‐phase microextraction (SPME) with microfluidics for the online mass spectrometric profiling of real‐time prodrug metabolism. (a) Schematic representation of the integrated microfluidic‐SPME‐LC‐MS module. The system illustrates the pneumatic control of wash (100% ACN), equilibration (0.1% FA), and elution (0.1% FA / 50% MeOH) solutions for automated online sample preparation using an embedded C18 ZipTip. (b) Photograph of the microfluidic device displaying the interconnected fluidic channels and LC‐MS interface tubing. Scale bar: 10 mm. (c,d) High‐resolution mass spectra of (c) the prodrug capecitabine (m/z 358.14) and (d) its intermediate metabolite 5′‐deoxy‐5‐fluorouridine (DFUR, m/z 245.05), detected in the HepaRG spheroid effluent after 48 h of treatment. (e, f) Mass spectra of (e) tamoxifen (m/z 372.23) and (f) its CYP‐mediated pharmacologically active metabolite, 4‐hydroxytamoxifen (4‐OHT, m/z 388.22), following a 48‐h incubation. (g) Extracted ion chromatograms (EIC) for 4‐OHT and tamoxifen, alongside the total ion chromatogram (TIC). (h,i) Mass spectrometric intensity profiles tracking the (h) depletion of tamoxifen and (i) sequential generation of 4‐OHT in the hepatocyte spheroids medium over a 48‐h period. (j,k) Corresponding time‐dependent quantitative concentration curves (µM) for (j) tamoxifen and (k) 4‐OHT in the hepatocyte spheroid medium over 48 h. Data points are connected by solid lines to highlight the metabolic trends. Data are presented as mean ± SEM (n = 3).

To profile prodrug efficiency and safety, the validation and assessment of the pharmacokinetic pathway of the prodrug metabolism and activation are essential. To date, there remains a significant challenge in integrating online mass spectrometry with OoC technology. We addressed this limitation by creating an integrated microfluidic‐mass spectrometry platform that enables real‐time monitoring of prodrug metabolism within our iMULTI‐chip system (Figure 4a,b). This platform features an automated online solid‐phase microextraction (SPME) module, utilizing a C18 stationary phase, which interfaced directly with liquid chromatography‐tandem mass spectrometry (LC‐MS/MS) analysis, allowing for automated sample preparation and analyte enrichment from the microfluidic effluent (Figure 4b). This configuration utilized a sophisticated valve‐controlled fluidic network with dedicated channels for wash solution, equilibration solution, and elution solution to maintain optimal chromatographic conditions and minimize matrix interference from the complex biological medium (see Figures S22 and S23 for detailed microfluidic SPME procedure; Video S6). Crucially, the data demonstrate the power of this method to profile real‐time metabolized prodrugs and their reactive intermediates, allowing for automated pharmaceutical kinetics and dynamics landscaping directly on the chip.

Following the administration of capecitabine to HepaRG spheroids within our microfluidic device, high‐resolution online MS analysis after a 48‐h incubation demonstrated significant metabolic conversion. We performed high‐resolution mass spectrometry analysis of standard precursor drugs and their metabolites (at a concentration of 300 µM), using negative ion mode for capecitabine and positive ion mode for tamoxifen. The recorded mass‐to‐charge (m/z) ratios for capecitabine (m/z 358.1407) and its enzymatically derived intermediate, 5′‐deoxy‐5‐fluorouridine (DFUR, m/z 245.0574), exhibited exceptional accuracy with negligible mass deviations (Figure 4c,d, Figure S24). This confirms the robust preservation of vital enzymatic activities, specifically cytidine deaminase and thymidine phosphorylase, within our dynamic 3D hepatic construct. Similarly, we investigated the biotransformation of tamoxifen, a clinically ubiquitous prodrug strictly dependent on cytochrome P450‐mediated hepatic metabolism. Mass spectrometric evaluations confidently identified both the parent compound, tamoxifen (m/z 372.2313), and its pharmacologically active metabolite, 4‐hydroxytamoxifen (4‐OHT, m/z 388.2260), further corroborating the preserved phase I metabolic capacity of the dynamic liver model (Figure 4e,f).

To precisely map the time‐dependent pharmacokinetics of tamoxifen metabolism, we established quantitative protocols. Extracted ion chromatograms (EIC) and total ion chromatograms (TIC) confirmed the chromatographic separation and high‐sensitivity detection of the target analytes over the elution period (Figure 4g). Standard calibration curves for both tamoxifen and 4‐OHT exhibited excellent linearity (*R* ^2^ =  0.9995 and *R* ^2^ =  0.9952, respectively), laying a reliable foundation for precise quantification (Figure S25). Leveraging this automated, non‐invasive setup, time‐course evaluations over 48 h revealed a progressive, time‐dependent decline in tamoxifen MS intensity and concentration within the liver medium (Figure 4h,i). Concomitantly, this depletion was mirrored by a steady kinetic accumulation of the active metabolite 4‐OHT (Figure 4j,k). From the unperturbed hepatic spheroids, the temporally‐resolved depletion of tamoxifen and the concomitant non‐linear accumulation of 4‐OHT (6, 24, 36 and 42 h) allowed us to determine the precise temporal window at which the active metabolite reaches therapeutically relevant concentrations. Collectively, these findings underscore the transformative utility of the integrated SPME‐LC‐MS platform. By enabling the continuous pharmacokinetic profiling of prodrugs and their intermediates, this system provides crucial mechanistic insights into metabolic activation pathways, effectively bridging the gap between in vitro tissue engineering and real‐time pharmacological evaluation.

### 2.5 iMULTI‐Chip for Prodrug Pharmacodynamic Evaluation

**FIGURE 5:**
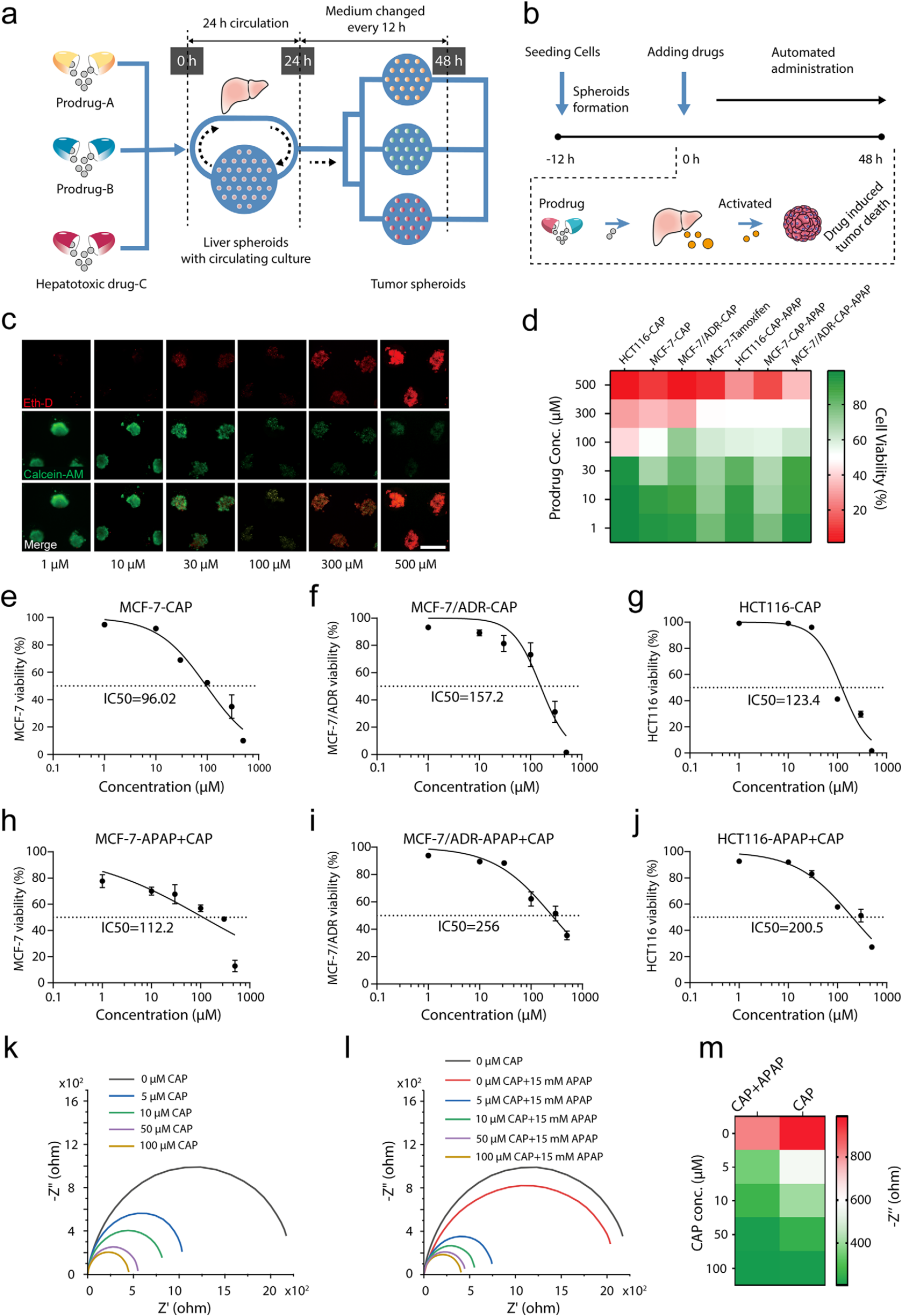
Evaluation of multi‐organ pharmacodynamics, prodrug efficacy, and real‐time hepatotoxicity monitoring on the iMULTI‐chip platform. (a) Schematic representation of the multi‐organ microfluidic configuration illustrating the experimental design: administration of prodrugs (capecitabine, tamoxifen) and hepatotoxic agent to liver spheroids under dynamic circulatory treatment, with subsequent exposure of metabolites to downstream tumor spheroid arrays. (b) Experimental timeline delineating sequential processes: initial cell seeding (−12 h), spheroid formation, drug administration (0 h), and continuous monitoring over 48 h, with inset illustrating the mechanistic progression from prodrug administration to hepatic metabolic activation and subsequent tumor cytotoxicity. (c) Representative confocal fluorescence images of target tumor spheroids stained with Calcein‐AM (live, green) and Ethidium Homodimer‐1 (dead, red) after exposure to varying concentrations (1–500 µM) of metabolically activated prodrugs in liver co‐culture. Scale bar: 100 µm. (d) Comprehensive heatmap of cancer cell viability across multiple cell lines (HCT116, MCF‐7, MCF‐7/ADR) in response to varying concentrations (1–500 µM) of capecitabine (CAP) alone or in combination with acetaminophen (APAP). (e–g) Dose‐response curves and calculated IC_50_ values for capecitabine monotherapy targeting (e) MCF‐7, (f) MCF‐7/ADR, and (g) HCT116 spheroids. (h–j) Dose‐response curve with calculated IC_50_ values for combined APAP + capecitabine treatment. (k,l) Nyquist plots derived from electrochemical impedance spectroscopy measuring real‐time secreted albumin levels under (k) CAP treatment alone and (l) CAP combined with 15 mM APAP, reflecting compromised hepatic function. (m) Heatmap of the real‐part impedance (− *Z*′′) comparing the dose‐dependent impact of CAP versus CAP +15 mM APAP on hepatocyte albumin secretion. Data represent mean ± SEM (n = 3 independent experiments).

In vitro study of normal and pathological physiology requires recapitulating the complex crosstalk between organ systems, particularly the integration of hepatic drug metabolism and subsequent tumor eradication within interconnected networks [58, 59, 60]. We next used our iMULTI‐chip system to explore the online analysis of liver‐dependent prodrug pharmacokinetics and pharmacodynamics. The integrated microfluidic platform enables sequential activation of prodrugs by hepatic spheroids followed by exposure of tumor spheroids to the resultant metabolites under physiologically relevant conditions (Figure 5a). In the system, drugs were administered through the inlet to the liver spheroids chamber to mimic the first pass metabolism. The experimental protocol involved the initial seeding and formation of tumor and liver spheroids (−12 h), followed by prodrug administration (0 h), automated medium circulation for 24 h, and subsequent delivery to the tumor spheroid chamber every 12 h (Figure 5b). This operation recapitulates the in vivo process where prodrugs undergo hepatic biotransformation before exerting therapeutic effects on distant tumor tissues.

To verify the prodrug efficacy screening capabilities of the platform, we conducted comprehensive dose‐response studies using capecitabine (CAP) across three different cancer cell types in spheroid form: MCF‐7, MCF‐7/ADR, and HCT116 cells. The tumor aggregates were grown with a separately controlled dynamic flow (not affected by the liver circulation flow) on the chip for a minimum of 24 h prior to drug exposure. At a certain time point, the chamber top membrane was removed to facilitate the standard staining procedure. Fluorescence viability staining reflects dose‐dependent cytotoxicity in tumor spheroids exposed to liver‐activated capecitabine, with marked cell death evident at concentrations of 100 µM or higher (Figure 5c,d). Quantitative viability assessment demonstrated differential sensitivity across cancer cell lines, with MCF‐7 breast cancer cells exhibiting the highest sensitivity to capecitabine (IC_50_ = 96.02 µM), while the multidrug‐resistant variant MCF‐7/ADR displayed significantly reduced susceptibility (IC_50_ = 157.2 µM, p < 0.01), confirming preserved resistance phenotypes within our microfluidic platform (Figure 5e,f). HCT116 colorectal cancer spheroids showed intermediate sensitivity (IC_50_ = 123.4 µM), the observed in vitro sensitivity trends are consistent with the expected performance of this cell line in studies of similar chemotherapeutic agents (Figure 5g).

To investigate potential drug‐drug interactions, a common clinical scenario that can drastically alter drug metabolism, we examined the combined effects of acetaminophen (APAP) co‐administration with capecitabine. Interestingly, APAP co‐exposure moderately increased the IC_50_ of capecitabine in MCF‐7 cells from 96.02 to 112.2 µM (Figure 5h), suggesting potential competitive inhibition of metabolic activation pathways. This effect was more pronounced in MCF‐7/ADR cells, where the IC_50_ increased from 157.2 to 256 µM (Figure 5i), and in HCT116 cells (IC_50_ increased from 123.4 to 200.5 µM) (Figure 5j). Comprehensive heatmap analysis across all experimental conditions and concentrations (1–500 µM) confirmed these findings, with the most substantial cytotoxicity observed with single‐agent treatments at high concentrations (≥300 µM) and relative protection conferred by APAP co‐administration, particularly in the resistant MCF‐7/ADR cell line (Figure 5d). This suppression of antineoplastic activity distinctly reflects the APAP‐induced impairment of hepatic enzymatic function, thereby diminishing the biotransformation of capecitabine into its active cytotoxic derivative.

To precisely validate this decline in liver function, we consequently performed a dose‐dependent electrochemical analysis of secreted albumin in the culture medium, serving as an online biomarker for hepatocyte viability and metabolic integrity. Electrochemical impedance spectroscopy (EIS), represented through Nyquist plots, demonstrated a sequential decrease in impedance (‐*Z*′′) corresponding to varying CAP concentrations, effectively mapping baseline liver function (Figure 5k). Conversely, the co‐administration of CAP and APAP resulted in drastically distinct impedance profiles and a substantially reduced overall charge transfer resistance, quantitatively confirming severe disruption of albumin secretion due to profound hepatotoxicity (Figure 5l). The hepatotoxic effects of APAP were further visualized through the heat map in Figure 5m. By seamlessly leveraging dual‐mode optical and electrochemical sensing, the iMULTI‐chip system enables high‐fidelity, real‐time assessment of drug‐induced side effects, offering a highly robust platform for advanced prodrug screening, personalized pharmacological profiling, and complex pathophysiological modeling.

## 3 Conclusion

The translation of prodrugs from discovery to the clinic is frequently hampered by the inability of conventional preclinical models to dynamically track enzymatic activation and off‐target toxicity. The iMULTI‐chip platform presented in this work represents a significant advancement in microphysiological systems by successfully integrating 3D multicellular architectures with multimodal, non‐invasive analytical capabilities. While previous OoC platforms have enabled multi‐organ interactions, they have largely lacked the sophisticated analytical integration necessary for comprehensive prodrug metabolism studies [26, 45, 61]. Our multi‐modular system overcomes these limitations through several key innovations: (i) strategic microwell array configurations that promote the development of metabolically competent 3D hepatic constructs; (ii) real‐time electrochemical impedance spectroscopy‐based immunosensing for quantitative assessment of hepatic function and drug‐induced toxicity; and (iii) automated solid‐phase microextraction coupled with mass spectrometry (SPME‐LC‐MS) in a seamless sample‐to‐spectrum pipeline, for non‐invasive longitudinal monitoring of pharmaceutical kinetics. This multimodal architecture enables simultaneous, automated, and non‐invasive profiling of both drug‐induced hepatotoxicity (via secreted albumin quantification) and prodrug metabolic conversion kinetics from the same on‐device tissue over extended culture periods, a capability that remains fundamentally unmet in conventional multi‐organ‐on‐a‐chip systems.

The validation studies with capecitabine and tamoxifen demonstrate the platform's unique capacity to characterize complex hepatic metabolic activation pathways and their subsequent impact on tumor response, addressing a critical gap in current in vitro drug testing paradigms. Particularly noteworthy is the system's ability to maintain physiologically relevant cellular functions while simultaneously performing multi‐parameter analyses without compromising tissue integrity, a feature essential for accurate recapitulation of prodrug metabolism. We demonstrated that this design enables precise control over fluid dynamics, which has been shown to significantly enhance metabolic maturation compared to static culture systems by proteomic landscaping. The proof‐of‐concept studies with capecitabine and tamoxifen unambiguously demonstrated preserved cytidine deaminase, thymidine phosphorylase, and cytochrome P450‐mediated enzymatic activities within the hepatic spheroids, enabling quantitative tracking of metabolic conversion with high mass accuracy and temporal resolution. Furthermore, the acetaminophen co‐administration attenuated capecitabine‐mediated antineoplastic activity across multiple tumor spheroid types (MCF‐7, MCF‐7/ADR, and HCT116) exemplifies the platform's capacity to dissect complex, clinically relevant pharmacological phenomena that are inaccessible to single‐organ or endpoint‐dependent systems.

Despite these advances, several limitations remain to be addressed. A significant challenge of the current platform is the incomplete recapitulation of the tumor microenvironment, particularly the absence of functional vasculature, stromal components, and immune cell interactions that play critical roles in drug response and resistance mechanisms [42]. Additionally, the current platform exhibits limitations in long‐term culture stability beyond 14 days, potentially restricting studies of chronic drug effects. The integration density of analytical modules, while advanced, still presents spatial constraints that limit simultaneous multi‐parameter monitoring across all tissue compartments.

Collectively, this integrated approach opens numerous avenues for advanced pharmaceutical research, including evaluation of patient‐specific responses using iPSC‐derived organoids, investigation of secondary organ toxicities resulting from hepatic metabolism (such as cardiotoxicity from metabolites), and high‐throughput assessment of complex therapeutic regimens with temporal and sequential administration protocols. Future iterations will focus on incorporating perfusable vascular networks using sacrificial printing techniques and integrating immune components to better mimic tumor‐immune interactions [62]. The development of miniaturized, multiplexed sensor arrays will enhance spatial resolution of metabolic monitoring. Furthermore, integration with machine learning algorithms for real‐time data analysis could enable predictive modeling of drug responses. By addressing these technical challenges and bridging the gap between tissue engineering and analytical chemistry within a single platform, the iMULTI‐chip establishes a new paradigm for mechanistic studies of drug metabolism and efficacy evaluation, potentially reducing reliance on animal models while increasing the predictive power of preclinical drug screening.

## 4 Experimental Section

### 4.1 Chip Fabrication

All microfluidic devices were fabricated with multilayer soft lithography. The manufacturing process for the liver‐tumor co‐culture microfluidic chip and mass spectrometry pre‐treatment chip includes the fabrication of molds and PDMS devices. The flow, control, and microwell array molds were patterned on separate silicon wafers by photolithography.

The silicon wafer was cleaned with acetone, isopropanol, and ethanol, respectively, blow‐dried, and baked on a hotplate (Torrey Pines Scientific, HP61A). The wafers were then baked at 200°C for 30 min to dehydrate them. After cooling down the wafers to room temperature, negative photoresist SU8‐2025 (MicroChem, U.S.A.) was spin‐coated (KW‐4A, Institute of Microelectronics of the Chinese Academy of Science, Beijing, China) at 500 rpm for 5 s and 3000 rpm for 40 s. The wafer rested on a flat surface for 10 min to ensure the uniformity of the photoresist. Then the wafer was baked at 65°C for 2 min and 95°C for 6 min and exposed to UV radiation for 25 s with a photolithography aligner (7 mW / cm^2^, BG‐401A, CETC, China), under a high‐resolution photo‐mask (MicroCAD photo‐mask, Shenzhen, China). Post‐exposure bake was then carried out on the hotplate for 1 min at 65°C and 95°C for 4 min, respectively. Then, the wafer was developed in SU8 developer (MicroChem, USA) and gently washed with isopropanol, followed by blow drying. After development, the mold was blow‐dried and baked on a hotplate at 115°C for 1 h and then ramped up to 200°C for 5 h, with 10°C/h. The height of the control layer channel was about 30 µm.

To fabricate the microwell array mold, negative photoresist (SU8‐2100, MicroChem, U.S.) was poured onto the wafer and spun at 500 rpm for 5 s and 900 rpm for 40 s. The wafer rested on a flat surface for 30 min to ensure the uniformity of the photoresist. After baking at 65°C for 10 min and 95°C for 120 min, the wafer was exposed to the UV light for 60 s, and baked again at 65°C for 5 min and 95°C for 30 min. The microwell array mold was developed and baked on a hotplate by ramping from 95°C to 180°C at 20°C/h for 5 h. The height of the control layer channel was about 300 µm.

The wafer of the flow‐layer mold was coated with positive photoresist (AZ4620, MicroChemicals GmbH) by spinning at 500 rpm for 5 s and 1000 rpm for 40 s, and baked at 110°C for 4 min. Then the wafer was exposed to UV radiation through a positive mask for 40 s and developed in AZ400K developer (DI water:developer = 2:1). The wafer was rinsed by blow drying and baked by ramping from 65°C to 220°C at 20°C/h for 9 h to reflow the positive photoresist. The height of the flow channels was about 15 µm. To facilitate PDMS releasing from mold, we subsequently salinized chip molds by trichloro (1H, 1H, 2H, 2H‐perfluorooctyl) silane (Sigma‐Aldrich) vapor for 1 h. The flow layer chip was designed in a push‐up manner. About 45 g of PDMS mixture (polydimethylsiloxane, RTV615, GE Advanced Materials) with a proper ratio (base agent: crosslinking agent = 5:1) was poured onto the mold wafer, and subsequently degassed. After being baked at 80 °C for 60 min, the cured PDMS slab was carefully cut and peeled from the mold. The chip was then trimmed into an appropriate size by using an art knife (OLFA, Japan). A 0.75 mm biopsy (WPI, Sarasota, USA) was used to punch holes for the inlets. In terms of the control mold, PDMS mixture with a ratio of 20:1 (monomer: crosslinking agent) was spin‐coated at 500 rpm for 5 s and 1600 rpm for 40 s and subsequently baked at 80 °C for 10 min. The flow layer was aligned to the thin control‐layer wafer under a stereo microscope (SMZ745T, NIKON, Japan), baked at 80°C for 2 h. The bonded layers were then carefully peeled off from the control wafer, and punch holes using 0.75 mm, 3 mm, and 4 mm biopsy punches (WPI, USA).

To fabricate the microwell array layer, the PDMS mixture (base: curing agent is 10:1) was spin‐coated at 500 rpm for 10 s and rested for 10 min at room temperature. After curing, carefully peel it off from the silicon wafer, align it with the bonded fluid‐control layer under a stereo microscope, and place the entire chip in an 80°C oven for overnight bonding. The mass spectrometry pre‐treatment chip was fabricated using the same method as the cell culture chip, except that its third layer consists of glass, and the bonding was achieved via plasma activation.

### 4.2 3D‐Printed Three‐Electrode Adapter

The adapter model, including parameters such as channel layout and dimensions, was designed using Cinema 4D software (Maxon). The digital model was uploaded to a 3D printer (Form3, Formlabs) via the software Preform. Formlabs' clear resin was used for printing the chip. Following initial cleaning, a 20 mL syringe was connected to each inlet and outlet port sequentially, and isopropyl alcohol was flushed through all internal channels a minimum of five times to eliminate residual uncured resin. After air drying for 30 min at room temperature, the chips were subjected to postcuring in a UV curing chamber (Form Cure, Formlabs) at 60°C for precisely 20–30 min to achieve optimal mechanical properties and chemical stability (Figure S14).

### 4.3 Fluid Actuation, Recirculation Formation, and Flow Rate Analysis

All the valves in our multilayer device were operated by the pneumatic solenoid valve (Pneumadyne, USA) and pneumatic array control system (FluidicLab, Shanghai, China) with programmable scripting automation [63]. Three valves arranged along a single fluidic channel constitute a peristaltic pump, which drives fluid circulation within the closed‐loop circuit. This setup enables the culture medium to continuously recirculate within a self‐contained loop, simulating peristaltic blood flow in vascular systems. Peristaltic pumping was achieved by sequential actuation of the three membrane valves in the pattern 101, 100, 110, 010, 011, 001, where 0 and 1 represent the open and closed states of the valves, respectively. To determine the recirculating flow rate, the displacement of 5 µm polystyrene microspheres within the fluid channel was tracked (Figure S5). Particle trajectories were analyzed using Fiji software [64]. To calculate the volumetric flow rate in the channel, the cross‐sectional area of the fluidic channel was also measured in Fiji. The volumetric flow rate, defined as the volume of solution passing through the channel per unit time, was determined to be 0.323 µL/s.

### 4.4 Cell Culture

The HepaRG cell line was generously provided by Dr. Weiping Ding's lab at the University of Science and Technology China. HepaRG, HCT116, and MCF‐7 cells were cultured in 90% Dulbecco's Modified Eagle Medium (DMEM, Gibco, USA), 10% FBS, and 1% P/S, while MCF‐7/ADR cells were maintained in RPMI‐1640 medium (Gibco, USA), 10% FBS, and 1% P/S. The culture medium was replaced every 2 days until the cells reached approximately 80%–90% confluence in a T25 flask. The MCF‐7/ADR cells were treated with 500 ng/ml of doxorubicin for at least 24 h prior to the experiment. The passage of the cells was performed using 0.25% trypsin–EDTA solution (Gibco, USA) and passaged at a 1:2 split ratio.

### 4.5 On‐Chip Spheroids Culture

In terms of the three‐dimensional cell spheroid culture, we adopted our previously published protocols to generate the multicellular spheroids with a microwell array structure [65]. Briefly, the 75% ethanol sterilized microfluidic chip was degassed in a vacuum desiccator and soaked overnight in PBS containing 2% (w/v) Pluronic F‐127 (Sigma). The chip was then rinsed with PBS to remove residual Pluronic solution. Different cells are seeded into the corresponding culture chambers, then centrifuged at 1000 rpm for 5 min. After centrifugation, the excess culture medium is aspirated and fresh medium is added. The system is then incubated overnight in the incubator for aggregation culture. Then the chip's control lines of the multiplexer control device were connected to pneumatic solenoid valves (Pneumadyne, Plymouth, USA) through tubing (Tygon, 0.060 inch outer, 0.020 inch inner diameter) and a proper luer‐lock adapter (Nordson MEDICAL, USA). The valves were controlled with an array control system (FluidicLab, Shanghai, China). Through visual confirmation, all the air bubbles were removed by pressurizing the fluid layer with a properly closed control line. To ensure smoother drug metabolism and transfer, a PDMS plug is inserted at the top of the cell culture chamber and connected to a gas control valve. The culture medium with or without the drug and the waste vials were also connected with the system. Once connected, the custom program delivers the desired solution to the liver chamber and other tumor spheroid chambers under a proper flow rate. The whole system was then put into an incubator with proper temperature (37 °C), humidity (100%), and CO_2_ (5%) composition to maintain cell culture.

### 4.6 Quantitative Real‐Time Polymerase Chain Reaction

Hepatic gene expression in HepaRG cells under varying culture conditions was analyzed following molecular characterization protocols. Spheroids were individually extracted from microwell structures via mouth pipetting using a 300 µm diameter capillary needle fabricated with a micropipette puller (PC‐10, Narishige, Japan). For each technical replicate, approximately 5–10 spheroids were directly transferred to 0.2 mL PCR tubes [51]. Total RNA isolation was performed using the RNeasy Mini kit (Qiagen) and subsequently purified following the manufacturer's manual. RNA quality and concentration were assessed spectrophotometrically using a NanoDrop One/OneC Microvolume UV–vis Spectrophotometer (Thermo Scientific, USA). Following isolation, isolated RNA was reverse transcribed using a QuantiTect reverse transcription kit (Qiagen). The gene‐specific oligonucleotide primers used for PCR amplification are listed in Table S1 (Supporting Information). To amplify the cDNA, 10 cycles of PCR were performed with target gene primers using a Takara kit (Takara Taq Hot Start version). After preamplification, cDNA was purified using the QIAquick PCR Purification Kit. Quantitative PCR was then employed to measure expression levels of specific hepatic metabolic markers, including CYP1A1, CYP2C19, CYP2C9, and Albumin. The qPCR reactions were conducted using SYBR qPCR Master Mix (TaqPro Universal, Vazyme, Nanjing) on a Real‐Time PCR System (q225, Kubo Technology, Beijing, China). Thermocycling conditions consisted of initial denaturation at 95°C for 10 min, followed by 40 cycles of denaturation (95°C, 10 s), annealing (65°C, 15 s), and extension (72°C, 45 s). Relative gene expression levels were quantified using the comparative threshold cycle method (‐ΔΔCt), with β‐actin serving as the endogenous reference for normalization.

### 4.7 Immunofluorescence Analysis

Cell spheroids cultured in microfluidic chips were first fixed with 4% paraformaldehyde at room temperature for 10 min, followed by permeabilization with 0.1% Triton X‐100 for 5 min. Subsequently, the samples were blocked with 1% bovine serum albumin (BSA) solution for 1 h. The spheroids were then incubated overnight at 4°C with primary antibodies. For hepatocyte spheroids, the following primary antibodies were used: mouse monoclonal anti‐HNF4‐α (AB41898, 1:100, Abcam), rabbit polyclonal anti‐ZO‐1 (ab221547, 1:100, Abcam), and anti‐albumin (MA529022, 1:100, Invitrogen). After primary antibody incubation, the samples were gently washed twice with TBST and incubated at room temperature for 1 h with the corresponding secondary antibodies: Alexa Fluor 488‐conjugated goat anti‐mouse IgG (A24877, 1:250, Invitrogen), Alexa Fluor 555‐conjugated donkey anti‐rabbit IgG (A24869, 1:250, Invitrogen), and Alexa Fluor 647‐conjugated goat anti‐rabbit IgG (ab150079, 1:250, Abcam). Nuclear staining was performed using DAPI (R37606, Invitrogen). To ensure homogeneous staining of the 3D spheroids, a 40 µm cell strainer (Falcon, Corning) was employed to collect the spheroids after each immersion step until the staining procedure was completed. The stained spheroids retained on the strainer were transferred into confocal dishes, gently overlaid with a coverslip, and imaged using an inverted microscope equipped with a laser scanning confocal module (Nikon AXR, Confocal, Japan). The acquired images were processed using NIS software.

### 4.8 Cell Viability Assay

In terms of cell viability, Calcein‐AM and Ethidium homodimer (Eth‐D) are used to assess cell viability. Briefly, spheroids were gently rinsed two to three times with DPBS, followed by incubation with a mixed dye solution containing 5 µM Calcein‐AM and 4 µM Eth‐D. The staining was performed in the dark for 20 min, after which the spheroids were gently rinsed twice with DPBS. The viability of the 3D spheroids was then observed using an epi‐fluorescence microscope (Nikon Eclipse Ti). For quantitative analysis, we estimate the fraction of live and dead cells by image analysis using Fiji software. The viability of spheroids was determined from the ratios of green/black and red/black areas after live/dead staining. Green and red fluorescence images were captured under identical conditions, allowing Fiji to quantify the area of each fluorescence channel separately. The following formula was used to calculate cell viability.

Cellviability%=AreaGreen/AreaBackgroundAreaRed/AreaBackground×100

### 4.9 Albumin and Urea Secretion Assay

To measure the albumin secretion from the HepaRG spheroids, the medium in the microwell array chamber was collected 24 h after the spheroids were generated. The level of albumin secreted into the culture medium was measured using a human albumin enzyme‐linked immunosorbent assay (ELISA) kit (RayBiotech, ELH‐Albumin, USA). Absorbance was read at 450 nm using a microplate reader (SpectraMax M4, Molecular Devices, Sunnyvale, CA, USA). Urea secretion was assessed using the same culture medium with a urea assay kit (Solarbio, BC1535, China), and absorbance was measured at 630 nm to obtain quantitative colorimetric results.

### 4.10 Sample Preparation for Proteomics

HepaRG cells were collected under different culture conditions, along with HepaRG spheroids treated with liver injury drug, acetaminophen, and capecitabine, into centrifuge tubes. The samples were centrifuged and washed twice with PBS to remove residual liquid. The tubes were then added to a lysis buffer (1% w/v SDC, 20 mM Tris‐HCl, pH 8.5) that had been pre‐cooled to 4°C. The samples were immediately heated at 95°C for 5 min to facilitate cell lysis and to inactivate endogenous proteins and phosphatases. After cooling and grinding in liquid nitrogen, the samples were centrifuged, and the supernatants were collected. Protein concentration was measured using the BCA method. Based on the BCA results, all samples were diluted to a uniform protein concentration of 1 µg/µL. Subsequently, the samples underwent reduction and alkylation. For this, 20 µL of 1 µg/µL protein sample was mixed with 1 µL of 100 mM TCEP and 1 µL of 400 mM CAA. The mixture was incubated at 45°C and 1500 rpm in the dark for 5 min. The samples were then digested by adding 0.8 µL of trypsin (0.5 µg/µL) at a substrate‐to‐enzyme ratio of 1:25–1:50 (wt/wt). The digestion was carried out overnight at 37°C. Finally, desalting was performed. To the digested protein samples, 200 µL 1% trifluoroacetic acid (in isopropanol) was added, and thoroughly mixed by vortex. The samples were then subjected to low‐speed centrifugation at 500 g for 10 s.

The supernatant was transferred to a desalting column, and centrifugation was performed at 3500 g for 3 min. The filtrate was collected and passed through the desalting column again, followed by centrifugation at 3500 g for an additional 3 min; the filtrate was then discarded. The desalting column was then washed with 100 µL of 1% trifluoroacetic acid (in isopropanol) and centrifuged at 3500 g for 2 min, discarding the filtrate. Subsequently, 100 µL of 0.2% trifluoroacetic acid/5% acetonitrile (solvent: HPLC‐grade water) was added to the column, and the centrifugation was repeated under the same conditions. The filtrate was discarded again, and 100 µL of 60% acetonitrile/25% ammonia water (solvent: HPLC‐grade water) was added. After centrifugation at 3500 g for 2 min, the eluate was collected, and the procedure was repeated once more. The two collected eluates were combined and vacuum‐dried at 45°C and 1600 rpm for 3–4 h [66].

### 4.11 LC‐MS/MS Analysis

After sample preparation, peptides were chromatographically separated on a home‐made reverse‐phase C18 analytical column (15 cm length, 100 µm inner diameter, packed with 1.9 µm C18 particles). Liquid chromatography‐tandem mass spectrometry (LC‐MS/MS) analysis was performed using a data‐independent acquisition (DIA) approach on an Ultimate 3000 nanoLC system (Thermo Scientific, USA) coupled with an Orbitrap Eclipse Tribrid mass spectrometer (Thermo Scientific, USA). The mobile phase A consisted of 0.1% formic acid, while mobile phase B was 0.1% formic acid in 95% acetonitrile. The flow rate was set to 0.3 µL/min. The gradient elution was performed over 60 min as follows: 0–4 min, 5% B; 4–5 min, 15% B; 5–34 min, 25% B; 34–49 min, 40% B; 49–50 min, 99% B; 50–60 min, 99% B. In positive ion mode, the ion source spray voltage was set to 2.3 kV. HCD fragmentation was employed, with a full width at half maximum (FWHM) set to 18 s. The MS1 resolution was set to 60000, with a scan range from 400 to 1200 m/z. The RF Lens was set at 60%, and the automatic gain control (AGC) target was 3e6, with a maximum ion injection time (Max IT) of 54 ms. The scan type was set to Profile. For MS2 (DIA) scans, the resolution was set to 120 000, with an isolation window of 15 m/z. HCD fragmentation was used with a collision energy of 32%. The scan range was set from 150 to 2000 m/z, with the RF Lens set at 50%, the AGC target at 1 × 10^6^, and the Maximum IT set to 250 ms. The scan type was also set to Profile.

### 4.12 Proteomics Data Analysis

Raw LC/MS data were analyzed using DIA‐NN software (version 2.2.0). Protein identification was performed using the UniProt human reference proteome database, employing the FASTA digest for library‐free search and a deep learning‐based spectral matching algorithm to enhance spectral accuracy. The proteomics identification parameters were as follows: digestion was performed using Trypsin/LysC, with one missed cleavage allowed; fixed modification was carbamidomethylation, and variable modifications included oxidation and N‐terminal methionine excision. The peptide length was restricted to 7–30 amino acid residues, with precursor ion charge ranging from 1 to 5, and the precursor m/z range was set from 300 to 1800. Fragment ion m/z range was set from 200 to 1800. False discovery rate (FDR) for peptide and protein identification was controlled at 1% or below. All experimental groups and blank control samples were processed in the same batch and analyzed uniformly. Protein abundance data were normalized using the median of the shared proteins, and missing values were imputed with the minimum value. Differentially expressed proteins were selected based on the following criteria: fold change (FC) outside the range 0.67 and 1.5, and a p‐value of less than 0.05.

### 4.13 Operation of the Online Solid‐Phase Microextraction Chip

The SPME microfluidic module was integrated with the hepatic cell culture chip via the main inlet. Multi‐step extraction was achieved through three auxiliary inlets: (i) organic wash solution (100% acetonitrile), (ii) acidic equilibration solution (0.1% formic acid), and (iii) elution solution (1% formic acid/50% methanol). Solid‐phase extraction was performed using an embedded ZIPTIP‐C18 pipette tip (Merck, ZTC185, USA), with pneumatic control applied through the auxiliary channels. The elution solvent is delivered to the liquid phase vial via a capillary with a diameter of 666 µm, using pneumatic pressure. Subsequently, mass spectrometry data acquisition is performed by LC‐MS. The specific SPME procedure was as follows: the pipette tip was first activated with 100% acetonitrile, aspirating and dispensing three times; then equilibrated with 0.1% formic acid, aspirating and dispensing three times; sample loading was performed by aspirating and dispensing three times; the tip was washed with 0.1% formic acid, aspirating and dispensing three times; finally, the sample was eluted with 1% formic acid/50% methanol, aspirating and dispensing three times.

### 4.14 PPM Calculation Steps in Mass Spectrometry

PPM (parts per million) is used to express the deviation between the theoretical mass and the measured mass. PPM errors are commonly used to assess the precision of the instrument and the accuracy of the analytical results. The following equation outline how to calculate the PPM error for a molecule:

$$\Delta m=\left|Measued\;Mass-Theoretical\;Mass\right|$$

$$PPM=\frac{\Delta m}{Theoretical\;Mass}\;\times 10^{6}$$

Theoretical Mass: This is the molecular mass calculated based on the molecular formula. For ion peaks, the theoretical mass typically refers to the molecular ion ([M‐H]^−^ or [M+H]^+^, etc.) mass, either subtracting or adding the mass of the corresponding hydrogen atom. Measured Mass: This is the ion peak mass (m/z) obtained by the mass spectrometer. High‐resolution mode is typically used to obtain accurate ion masses. Generally, the smaller the PPM error, the more accurate the mass spectrometry analysis. A PPM value typically less than 5 is considered to have high precision and reliability.

### 4.15 Electrochemical Detection

The 3D‐printed electrochemical module was connected via the top tubing to the outlet of the hepatic chamber in the cell culture chip, with a side PBS inlet/outlet port enabling automated sampling and electrochemical detection. The affinity‐based biosensor employed in this study was based on a screen‐printed electrode (Poten, China), comprising a reference electrode (RE), a counter electrode (CE), and a gold working electrode (WE). Electrode functionalization followed the previously proposed strategy [67]. We use an electrochemical process to clean the electrodes. First, cyclic voltammetry (CV) was conducted in a 10 mM H_2_SO_4_ solution, followed by rinsing in PBS. Second, CV was performed in a 50 mM K_3_Fe(CN)_6_ solution for further cleaning. The gold WE surface was initially incubated in 10 mM 11‐mercaptoundecanoic acid (11‐MUA) solution for 1 h, followed by 30 min incubation in an EDC/NHS crosslinking solution (50 mM each dissolved in 50 mM citric acid buffer at PH 4.5) to convert 11‐MUA carboxyl groups into NHS esters, enabling coupling with primary amines in streptavidin.10 µg/mL Streptavidin was subsequently incubated for 1 h to form a uniform molecular pattern on the WE surface, after which, 10 µg/mL biotinylated anti‐human serum albumin antibody (ab24207, abcam, USA) was immobilized via strong biotin‐SPV interactions. A blocking step was applied to enhance sensor selectivity by incubating fresh culture medium containing bovine serum albumin (BSA) to eliminate nonspecific protein binding. Detection and quantification of protein biomarkers using biotinylated capture probes were performed via label‐free electrochemical methods by monitoring changes in interfacial electron transfer kinetics of [Fe(CN)_6_]^4‐/3−^ after biorecognition. Electrochemical impedance spectroscopy (EIS) was measured after each sample incubation to determine electrode impedance, with results presented as Nyquist plots (CHI760E, CH Instruments Ins). The real and imaginary components corresponded to semicircular and linear regions, distinguishing charge‐transfer‐limited from diffusion‐limited processes. Progressive antigen capture on the WE surface blocked the redox probe, resulting in a gradual increase in Rct values. Standard albumin concentrations were 0.5, 5, 10, 50, 100, 200, and 500 ng/mL. For actual samples, albumin levels were measured from 3D hepatic spheroids subjected to acetaminophen‐induced injury at concentrations of 1, 5, 10, 15, 20, 40, and 80 mM.

### 4.16 Liver Injury Model Construction and Electrochemical Detection

The HepaRG cell suspension is inoculated into pre‐treated microfluidic chips and centrifuged at 1000 rpm for 5 min. The chips are then incubated in a cell incubator for 24 h to allow for the formation of cell spheroid. To induce liver injury, the cells are perfused with acetaminophen (APAP) at varying concentrations (0.5 mM, 1 mM, 10 mM, 15 mM, 20 mM, 40 mM, and 80 mM) for 12–24 h. Following the establishment of the liver injury model, the metabolized culture medium is delivered to the electrochemical detection module via an automated device. The detection process proceeds as follows: the culture medium is incubated on the surface of the working electrode at room temperature for 1 h. The electrode surface is rinsed repeatedly with PBS. After adding 50 mM K_3_Fe(CN)_6_ as a redox probe. The impedance is measured across a frequency range of 0.1 to 100 000 Hz.

### 4.17 Quantitative Mass Spectrometry Analysis of Prodrug Metabolism

Desalting of the prodrug samples was performed using the ZipTip C18 solid‐phase microextraction method. To establish the calibration curves, tamoxifen was prepared at concentrations of 0.001, 0.01, 0.1, 1, 10, and 100 µM, while 4‐OHT was prepared at 0.001, 0.01, 0.1, 1, and 10 µM. Plot fitting was performed via simple linear regression using GraphPad Prism 9 software. For the metabolism assays, HepaRG cell spheroid were treated with 300 µM tamoxifen. At specified time intervals (6, 24, 36, and 42 h), automated extraction, desalting, and mass spectrometry analysis were conducted online. Both tamoxifen and 4‐OHT were detected using the positive ion mode. The actual metabolic concentrations of the analytes were subsequently calculated based on their respective standard calibration curves.

### 4.18 Statistical Analysis

All statistical analyses and data visualizations were performed using GraphPad Prism 9 (GraphPad Software, San Diego, CA, USA). Data are presented as the mean ± standard error of the mean (SEM) from at least three independent experiments (n = 3), unless otherwise specified. For multi‐group comparisons, statistical significance was determined using one‐way analysis of variance (ANOVA) followed by Tukey's post‐hoc test. Significance levels are denoted as follows: ^*^ *p* < 0.05, ^**^ *p* < 0.01, ^***^ *p* < 0.001, ^***^ *p* < 0.0001, ns indicates no significant difference (*p* > 0.05). For quantitative assays and drug toxicity screening, calibration curves were constructed using simple linear regression, while half‐maximal inhibitory concentration (IC_50_) values were calculated via non‐linear regression (four‐parameter logistic model). Electrochemical impedance spectroscopy (EIS) data were processed and fitted using the equivalent circuit analysis tool in Origin 2018. The relationship between cell viability and electrochemical impedance (‐Z'') was evaluated using Pearson correlation analysis and simple linear regression. The strength of the association was quantified by the Pearson correlation coefficient (r) and the coefficient of determination (R^2^). For proteomics, raw LC‐MS/MS data were processed using DIA‐NN software (version 2.2.0, University of Cambridge, UK). Differentially expressed proteins were selected based on the following criteria: fold change (FC) exceeding 0.67 and 1.5, and a *p*‐value of less than 0.05.

## Funding

National Natural Science Foundation of China (22174007, 22127805 and 22476223), Beijing Outstanding Young Scientist Program (No. BJJWZYJH01201910005017).

## Conflicts of Interest

The authors declare no conflict of interest.

## Supporting information




**Supporting File 1**: advs75056‐sup‐0001‐SuppMat.docx.

**Supporting File 2**: advs75056‐sup‐0002‐Video S1.mp4.

**Supporting File 3**: advs75056‐sup‐0003‐Video S2.mp4.

**Supporting File 4**: advs75056‐sup‐0004‐Video S3.mp4.

**Supporting File 5**: advs75056‐sup‐0005‐Video S4.mp4.

**Supporting File 6**: advs75056‐sup‐0006‐Video S5.mp4.

**Supporting File 7**: advs75056‐sup‐0007‐Video S6.mp4.

## Data Availability

The data that support the findings of this study are available in the supplementary material of this article.

## References

[1] M. Hay , D. W. Thomas , J. L. Craighead , C. Economides , and J. Rosenthal , “Clinical Development Success Rates for Investigational Drugs,” Nature Biotechnology 32 (2014): 40–51, 10.1038/nbt.2786. 24406927

[2] C. H. Wong , K. W. Siah , and A. W. Lo , “Estimation of Clinical Trial Success Rates and Related Parameters,” Biostatistics (Oxford, England) 20 (2019): 273–286, 10.1093/biostatistics/kxx069. 29394327 PMC6409418

[3] S. Bahtiri , T. M. S. Hagens , B. Van De Water , and M. Niemeijer , “Mechanism‐Based Drug Safety Testing Using Innovative In Vitro Liver Models: From DILI Prediction to Idiosyncratic DILI Liability Assessment,” Expert Opinion on Drug Metabolism & Toxicology 21 (2025): 769–787, 10.1080/17425255.2025.2516051. 40474568

[4] J. S. MacDonald and R. T. Robertson , “Toxicity Testing in the 21st Century: A View From the Pharmaceutical Industry,” Toxicological Sciences 110 (2009): 40–46, 10.1093/toxsci/kfp088. 19435982

[5] R. J. Andrade , N. Chalasani , E. S. Björnsson , et al., “Drug‐Induced Liver Injury,” Nature Reviews Disease Primers 5 (2019): 58, 10.1038/s41572-019-0105-0. 31439850

[6] J. Rautio , N. A. Meanwell , L. Di , and M. J. Hageman , “The Expanding Role of Prodrugs in Contemporary Drug Design and Development,” Nature Reviews Drug Discovery 17 (2018): 559–587, 10.1038/nrd.2018.46. 29700501

[7] Z. Fralish , A. Chen , S. Khan , P. Zhou , and D. Reker , “The Landscape of Small‐Molecule Prodrugs,” Nature Reviews Drug Discovery 23 (2024): 365–380, 10.1038/s41573-024-00914-7. 38565913

[8] D. E. Ingber , “Human Organs‐on‐chips for Disease Modelling, Drug Development and Personalized Medicine,” Nature Reviews Genetics 23 (2022): 467–491, 10.1038/s41576-022-00466-9. PMC8951665 35338360

[9] L. A. Low , C. Mummery , B. R. Berridge , C. P. Austin , and D. A. Tagle , “Organs‐On‐Chips: Into the next Decade,” Nature Reviews Drug Discovery 20 (2021): 345–361, 10.1038/s41573-020-0079-3. 32913334

[10] M. Wadman , “FDA no Longer Has to Require Animal Testing for New Drugs,” Science 379 (2023): 127–128, 10.1126/science.adg6276. 36634170

[11] A. Loewa , J. J. Feng , and S. Hedtrich , “Human Disease Models in Drug Development,” Nature Reviews Bioengineering 1 (2023): 545–559, 10.1038/s44222-023-00063-3. PMC10173243 37359774

[12] J. F. Feitor , L. C. Brazaca , A. M. Lima , et al., “Organ‐on‐a‐Chip for Drug Screening: A Bright Future for Sustainability? A Critical Review,” ACS Biomaterials Science & Engineering 9 (2023): 2220–2234, 10.1021/acsbiomaterials.2c01454. 37014814

[13] J. Yan , Z. Li , J. Guo , S. Liu , and J. Guo , “Organ‐on‐a‐Chip: A New Tool for In Vitro Research,” Biosensors and Bioelectronics 216 (2022): 114626, 10.1016/j.bios.2022.114626. 35969963

[14] Y. Zhu , K. Mandal , A. L. Hernandez , et al., “State of the Art in Integrated Biosensors for Organ‐On‐A‐Chip Applications,” Current Opinion in Biomedical Engineering 19 (2021): 100309. 37206309 10.1016/j.cobme.2021.100309 PMC10193909

[15] A. Skardal , S. V. Murphy , M. Devarasetty , et al., “Multi‐tissue Interactions in an Integrated Three‐tissue Organ‐on‐a‐chip Platform,” Scientific Reports 7 (2017): 8837, 10.1038/s41598-017-08879-x. 28821762 PMC5562747

[16] P. Deng , K. Cui , Y. Shi , et al., “Fluidic Flow Enhances the Differentiation of Placental Trophoblast‐Like 3D Tissue From hiPSCs in a Perfused Macrofluidic Device,” Frontiers in Bioengineering and Biotechnology 10 (2022): 907104, 10.3389/fbioe.2022.907104. 35845423 PMC9280037

[17] C. M. Leung , P. De Haan , K. Ronaldson‐Bouchard , et al., “A Guide to the Organ‐On‐A‐Chip,” Nature Reviews Methods Primers 2 (2022): 33.

[18] M. A. M. Subbaiah , J. Rautio , and N. A. Meanwell , “Prodrugs as Empowering Tools in Drug Discovery and Development: Recent Strategic Applications of Drug Delivery Solutions to Mitigate Challenges Associated With Lead Compounds and Drug Candidates,” Chemical Society Reviews 53 (2024): 2099–2210, 10.1039/D2CS00957A. 38226865

[19] R. Prantil‐Baun , R. Novak , D. Das , M. R. Somayaji , A. Przekwas , and D. E. Ingber , “Physiologically Based Pharmacokinetic and Pharmacodynamic Analysis Enabled by Microfluidically Linked Organs‐on‐Chips,” Annual Review of Pharmacology and Toxicology 58 (2018): 37–64, 10.1146/annurev-pharmtox-010716-104748. 29309256

[20] K. Brandauer , S. Schweinitzer , A. Lorenz , et al., “Advances of Dual‐Organ and Multi‐Organ Systems for Gut, Lung, Skin and Liver Models in Absorption and Metabolism Studies,” Lab on a Chip 25 (2025): 1384–1403, 10.1039/D4LC01011F. 39973270

[21] M. I. Khot , M. A. Levenstein , G. N. De Boer , et al., “Characterising a PDMS Based 3D Cell Culturing Microfluidic Platform for Screening Chemotherapeutic Drug Cytotoxic Activity,” Scientific Reports 10 (2020): 15915, 10.1038/s41598-020-72952-1. 32985610 PMC7522244

[22] T. Tao , P. Deng , Y. Wang , et al., “Microengineered Multi‐Organoid System From hiPSCs to Recapitulate Human Liver‐Islet Axis in Normal and Type 2 Diabetes,” Advanced Science 9 (2022): 2103495, 10.1002/advs.202103495. 34951149 PMC8844474

[23] Y. Du , Y. Wang , Q. Bao , et al., “Personalized Vascularized Tumor Organoid‐on‐a‐Chip for Tumor Metastasis and Therapeutic Targeting Assessment,” Advanced Materials 37 (2025): 2412815, 10.1002/adma.202412815. 39726096

[24] D. N. Tavakol , T. R. Nash , Y. Kim , et al., “Modeling the Effects of Protracted Cosmic Radiation in a Human Organ‐on‐Chip Platform,” Advanced Science 11 (2024): 2401415, 10.1002/advs.202401415. 38965824 PMC11558103

[25] G. Vunjak‐Novakovic , K. Ronaldson‐Bouchard , and M. Radisic , “Organs‐on‐a‐Chip Models for Biological Research,” Cell 184 (2021): 4597–4611, 10.1016/j.cell.2021.08.005. 34478657 PMC8417425

[26] K. Ronaldson‐Bouchard , D. Teles , K. Yeager , et al., “A Multi‐Organ Chip With Matured Tissue Niches Linked by Vascular Flow,” Nature Biomedical Engineering 6 (2022): 351–371, 10.1038/s41551-022-00882-6. PMC9250010 35478225

[27] B. Zhang , A. Korolj , B. F. L. Lai , and M. Radisic , “Advances in Organ‐on‐a‐Chip Engineering,” Nature Reviews Materials 3 (2018): 257–278, 10.1038/s41578-018-0034-7.

[28] F. Yin , X. Zhang , L. Wang , et al., “HiPSC‐Derived Multi‐Organoids‐on‐Chip System for Safety Assessment of Antidepressant Drugs,” Lab on a Chip 21 (2021): 571–581. 33319899 10.1039/d0lc00921k

[29] F. Gökçe , A. Kaestli , C. Lohasz , et al., “Microphysiological Drug‐Testing Platform for Identifying Responses to Prodrug Treatment in Primary Leukemia,” Advanced Healthcare Materials 12 (2023): 2202506. 36651229 10.1002/adhm.202202506 PMC11469234

[30] G. Lee , H. Kim , J. Y. Park , et al., “Generation of Uniform Liver Spheroids From human Pluripotent Stem Cells for Imaging‐Based Drug Toxicity Analysis,” Biomaterials 269 (2021): 120529, 10.1016/j.biomaterials.2020.120529. 33257114

[31] M. Lucchetti , K. O. Aina , L. Grandmougin , et al., “An Organ‐on‐Chip Platform for Simulating Drug Metabolism Along the Gut–Liver Axis,” Advanced Healthcare Materials 13 (2024): 2303943, 10.1002/adhm.202303943. 38452399

[32] J. Zhang , J. Wu , H. Li , Q. Chen , and J.‐M. Lin , “An In Vitro Liver Model on Microfluidic Device for Analysis of Capecitabine Metabolite Using Mass Spectrometer as Detector,” Biosensors and Bioelectronics 68 (2015): 322–328, 10.1016/j.bios.2015.01.013. 25599844

[33] C. W. McAleer , C. J. Long , D. Elbrecht , et al., “Multi‐organ System for the Evaluation of Efficacy and off‐target Toxicity of Anticancer Therapeutics,” Science Translational Medicine 11 (2019): aav1386, 10.1126/scitranslmed.aav1386. 31217335

[34] Y. Hou , X. Ai , L. Zhao , et al., “An Integrated Biomimetic Array Chip for High‐throughput co‐culture of Liver and Tumor Microtissues for Advanced Anticancer Bioactivity Screening,” Lab on a Chip 20 (2020): 2482–2494, 10.1039/D0LC00288G. 32542294

[35] S. A. P. Rajan , J. Aleman , M. Wan , et al., “Probing Prodrug Metabolism and Reciprocal Toxicity With an Integrated and Humanized Multi‐tissue Organ‐on‐a‐chip Platform,” Acta Biomaterialia 106 (2020): 124–135, 10.1016/j.actbio.2020.02.015. 32068138 PMC11083435

[36] F. Jiang , Y. Zhang , G. Fang , et al., “Intravasation‐On‐µDevice (INVADE): Engineering Dynamic Vascular Interfaces to Study Cancer Cell Intravasation,” Advanced Materials 37 (2025): 2501466, 10.1002/adma.202501466. 40223399 PMC12232236

[37] Y. Wang , H. Wang , P. Deng , et al., “In Situ Differentiation and Generation of Functional Liver Organoids From Human iPSCs in a 3D Perfusable Chip System,” Lab on a Chip 18 (2018): 3606–3616, 10.1039/C8LC00869H. 30357207

[38] S. Ya , W. Ding , S. Li , et al., “On‐Chip Construction of Liver Lobules With Self‐Assembled Perfusable Hepatic Sinusoid Networks,” ACS Applied Materials & Interfaces 13 (2021): 32640–32652, 10.1021/acsami.1c00794. 34225454

[39] M. Wang , Y. Sasaki , R. Sakagami , et al., “Perfluoropolyether‐Based Gut‐Liver‐on‐a‐Chip for the Evaluation of First‐Pass Metabolism and Oral Bioavailability of Drugs,” ACS Biomaterials Science & Engineering 10 (2024): 4635–4644, 10.1021/acsbiomaterials.4c00605. 38822812

[40] K.‐J. Jang , M. A. Otieno , J. Ronxhi , et al., “Reproducing human and Cross‐Species Drug Toxicities Using a Liver‐Chip,” Science Translational Medicine 11 (2019): aax5516, 10.1126/scitranslmed.aax5516. 31694927

[41] L. Ewart , A. Apostolou , S. A. Briggs , et al., “Performance Assessment and Economic Analysis of a Human Liver‐Chip for Predictive Toxicology,” Communications Medicine 2 (2022): 154, 10.1038/s43856-022-00209-1. 36473994 PMC9727064

[42] D. Choi , A. M. Gonzalez‐Suarez , M. G. Dumbrava , et al., “Microfluidic Organoid Cultures Derived From Pancreatic Cancer Biopsies for Personalized Testing of Chemotherapy and Immunotherapy,” Advanced Science 11 (2024): 2303088, 10.1002/advs.202303088. 38018486 PMC10837378

[43] J. Dornhof , J. Kieninger , H. Muralidharan , J. Maurer , G. A. Urban , and A. Weltin , “Microfluidic Organ‐on‐Chip System for Multi‐Analyte Monitoring of Metabolites in 3D Cell Cultures,” Lab on a Chip 22 (2022): 225–239, 10.1039/D1LC00689D. 34851349

[44] N. Jiang , G. Ying , Y. Yin , et al., “A Closed‐Loop Modular Multiorgan‐on‐Chips Platform for Self‐Sustaining and Tightly Controlled Oxygenation,” Proceedings of the National Academy of Sciences 121 (2024): 2413684121, 10.1073/pnas.2413684121. PMC11588096 39541351

[45] Y. S. Zhang , J. Aleman , S. R. Shin , et al., “Multisensor‐integrated Organs‐on‐chips Platform for Automated and Continual in Situ Monitoring of Organoid Behaviors,” Proceedings of the National Academy of Sciences 114 (2017): E2293–E2302. 10.1073/pnas.1612906114 PMC5373350 28265064

[46] K. Zhang , J. Xi , Y. Wang , et al., “A Microfluidic Chip‐Based Automated System for Whole‐Course Monitoring the Drug Responses of Organoids,” Analytical Chemistry 96 (2024): 10092–10101, 10.1021/acs.analchem.4c02075. 38833634

[47] Z. Izadifar , B. Charrez , M. Almeida , et al., “Organ Chips With Integrated Multifunctional Sensors Enable Continuous Metabolic Monitoring at Controlled Oxygen Levels,” Biosensors and Bioelectronics 265 (2024): 116683, 10.1016/j.bios.2024.116683. 39213819 PMC11391946

[48] M. A. Unger , H.‐P. Chou , T. Thorsen , A. Scherer , and S. R. Quake , “Monolithic Microfabricated Valves and Pumps by Multilayer Soft Lithography,” Science 288 (2000): 113–116, 10.1126/science.288.5463.113. 10753110

[49] M. J. Rupar , H. M. Hanson , B. L. Botlick , et al., “Translation of a Human‐Based Malaria‐on‐a‐Chip Phenotypic Disease Model for In Vivo Applications,” Advanced Science 12 (2025): 05206, 10.1002/advs.202505206. PMC12520463 40686419

[50] X. Wang , X. Liu , K. Li , et al., “A Microgel–Hydrogel Hybrid for Functional Compensation and Mechanical Stability in 3D Printed Cell‐Dense Vascularized Liver Tissue,” Advanced Materials 37 (2025): 2413940. 10.1002/adma.202413940 40223341

[51] L. Zhao , L. Wang , J. Huang , et al., “Label‐Free Imaging of Mesenchymal Stem Cell Spheroid Differentiation With Flexible‐Probe SECM and a Microfluidic Device,” Analytical Chemistry 96 (2024): 13473–13481, 10.1021/acs.analchem.4c01637. 39122667

[52] E. Moradi , S. Jalili‐Firoozinezhad , and M. Solati‐Hashjin , “Microfluidic Organ‐on‐a‐Chip Models of Human Liver Tissue,” Acta Biomaterialia 116 (2020): 67–83, 10.1016/j.actbio.2020.08.041. 32890749

[53] C. Ma , Y. Peng , H. Li , and W. Chen , “Organ‐on‐a‐Chip: A New Paradigm for Drug Development,” Trends in Pharmacological Sciences 42 (2021): 119–133, 10.1016/j.tips.2020.11.009. 33341248 PMC7990030

[54] R. Riahi , S. A. M. Shaegh , M. Ghaderi , et al., “Automated Microfluidic Platform of Bead‐Based Electrochemical Immunosensor Integrated With Bioreactor for Continual Monitoring of Cell Secreted Biomarkers,” Scientific Reports 6 (2016): 24598, 10.1038/srep24598. 27098564 PMC4838915

[55] Z. Rezaei , A. Navarro Torres , D. Ge , et al., “Noninvasive and Continuous Monitoring of On‐Chip Stem Cell Osteogenesis Using a Reusable Electrochemical Immunobiosensor,” ACS Sensors 9 (2024): 2334–2345. 38639453 10.1021/acssensors.3c02165

[56] T. N. H. Nguyen , L. F. Horowitz , T. Krilov , et al., “Label‐free, Real‐Time Monitoring of Cytochrome C Drug Responses in Microdissected Tumor Biopsies With a Multi‐well Aptasensor Platform,” Science Advances 10 (2024): adn5875, 10.1126/sciadv.adn5875. PMC11378948 39241078

[57] R. F.‐X. Tomasi , S. Sart , T. Champetier , and C. N. Baroud , “Individual Control and Quantification of 3D Spheroids in a High‐Density Microfluidic Droplet Array,” Cell Reports 31 (2020): 107670, 10.1016/j.celrep.2020.107670. 32460010 PMC7262598

[58] L. A. Milton , S. Kasetsirikul , J. A. Catano , et al., “Building multiple microenvironmental niches using a customizable 3D printed well insert,” Lab on a Chip 25 (2025): 5875–5893. 41048144 10.1039/d5lc00753d

[59] W. Hu , H.‐P. Bei , H. Jiang , et al., “DLM–GelMA/tumor Slice Sandwich Structured Tumor on a Chip for Drug Efficacy Testing,” Lab on a Chip 24 (2024): 3718–3727, 10.1039/D4LC00278D. 38953554

[60] G. Wu , J. Wu , Z. Li , et al., “Development of Digital Organ‐on‐a‐chip to Assess Hepatotoxicity and Extracellular Vesicle‐based Anti‐liver Cancer Immunotherapy,” Bio‐Design and Manufacturing 5 (2022): 437–450, 10.1007/s42242-022-00188-1.

[61] B. Schuster , M. Junkin , S. S. Kashaf , et al., “Automated Microfluidic Platform for Dynamic and Combinatorial Drug Screening of Tumor Organoids,” Nature Communications 11 (2020): 5271, 10.1038/s41467-020-19058-4. PMC7573629 33077832

[62] H. Liu , E. Noguera‐Ortega , X. Dong , et al., “A Tumor‐on‐a‐Chip for In Vitro Study of CAR‐T Cell Immunotherapy in Solid Tumors,” Nature Biotechnology (2025), 10.1038/s41587-025-02845-z. 41107533

[63] K. Brower , R. R. Puccinelli , C. J. Markin , et al., “An Open‐source, Programmable Pneumatic Setup for Operation and Automated Control of Single‐ and Multi‐layer Microfluidic Devices,” HardwareX 3 (2018): 117–134, 10.1016/j.ohx.2017.10.001. 30221210 PMC6136661

[64] J. Schindelin , I. Arganda‐Carreras , E. Frise , et al., “Fiji: An Open‐Source Platform for Biological‐Image Analysis,” Nature Methods 9 (2012): 676–682, 10.1038/nmeth.2019. 22743772 PMC3855844

[65] L. Zhao , Y. Liu , Y. Liu , M. Zhang , and X. Zhang , “Microfluidic Control of Tumor and Stromal Cell Spheroids Pairing and Merging for Three‐Dimensional Metastasis Study,” Analytical Chemistry 92 (2020): 7638–7645, 10.1021/acs.analchem.0c00408. 32374153

[66] N. A. Kulak , G. Pichler , I. Paron , N. Nagaraj , and M. Mann , “Minimal, Encapsulated Proteomic‐Sample Processing Applied to Copy‐Number Estimation in Eukaryotic Cells,” Nature Methods 11 (2014): 319–324, 10.1038/nmeth.2834. 24487582

[67] J. Aleman , T. Kilic , L. S. Mille , S. R. Shin , and Y. S. Zhang , “Microfluidic Integration of Regeneratable Electrochemical Affinity‐based Biosensors for Continual Monitoring of Organ‐on‐a‐Chip Devices,” Nature Protocols 16 (2021): 2564–2593, 10.1038/s41596-021-00511-7. 33911259

