# Supplementary material for: Multimodal Multiorgan‐on‐a‐Chip Platform for Probing Liver‐Tumor Interactions and Advancing Prodrug Screening
Source: Adv Sci (Weinh). 2026 Apr 7;13(34):e00040. doi: 10.1002/advs.202600040 (PMC13285169; doi:10.1002/advs.202600040)
Supplement: Supplementary file 1 — Supporting File 1: advs75056‐sup‐0001‐SuppMat.docx. [file ADVS-13-e00040-s004.docx]

Supporting Information

**A Multimodal Multiorgan-on-a-chip platform for probing Liver-Tumor Interactions and Advancing Prodrug Screening**

*Dan Wang, Yisong Huang, Weijian Zhao, Tian Chen, Siyu Bai, Denghui Guo, Mengying Wang, Yaoyao Zhao, Liang Zhao*, Guangsheng Guo*, Xiayan Wang**

*State Key Laboratory of Materials Low-Carbon Recycling, Beijing Key Laboratory of Cardiopulmonary-Cerebral Resuscitation Innovation and Translation, Center of Excellence for Environmental Safety and Biological Effects, Department of Chemistry, College of Chemistry and Life Science, Beijing University of Technology, Beijing, China 100124*

*Emails:*

*Liang Zhao: zhaoliang@bjut.edu.cn*

*Guangsheng Guo: gsguo@bjut.edu.cn*

*Xiayan Wang:* [*xiayanwang@bjut.edu.cn*](mailto:xiayanwang@bjut.edu.cn)

**Table of Contents**

Figure S1. Design and fabrication of the multi-organ microfluidic device. 4

Figure S2. Schematic illustration of the fabrication and assembly of the multi-organ microfluidic PDMS chip. 5

Figure S3. Design and characterization of the multi-organ microfluidic device. 6

Figure S4. Schematic diagram of an integrated automated microfluidic system. 7

Figure S5. Principle of closed-loop recirculating fluid actuation and flow rate detection. 8

Figure S6. Immunofluorescence micrographs of 2D culture conditions. 9

Figure S7. Manual selection of 3D liver cell spheroids using a glass pipette on the multi-organ microfluidic chip. 10

Figure S8. Venn diagram illustrates the overlap of differentially expressed proteins between the different culture conditions. 11

Figure S9. KEGG pathway enrichment analysis of differentially expressed proteins in hepatocyte spheroids cultured under different conditions. 12

Figure S10. Gene ontology (GO) enrichment analysis of differentially expressed proteins in hepatocyte spheroids under different culture conditions. 13

Figure S11. Sankey diagram illustrating differentially expressed proteins in hepatocyte spheroids cultured under different conditions. 14

Figure S12. Reactome pathway enrichment analysis of differentially expressed proteins in hepatocyte spheroids under different culture conditions. 15

Figure S13. Wikipathways enrichment analysis of differentially expressed proteins in hepatocyte spheroids under different culture conditions. 16

Figure S14. Photograph of the three-electrode system interfaced with the 3D-printed adapter. 17

Figure S15. Functionalization of the three-electrode system and detection of albumin standard. 18

Figure S16. Proteomic profiling of 3D hepatocyte spheroids exposed to APAP, CAP, or control. 19

Figure S17. GO enrichment analysis of proteomic data from 3D hepatocyte spheroids exposed to APAP, CAP, or control. 20

Figure S18. KEGG pathway enrichment analysis of proteomic data from 3D hepatocyte spheroids treated with APAP or CAP compared with control. 21

Figure S19. Reactome pathway enrichment analysis of proteomic data from 3D hepatocyte spheroids treated with APAP or CAP compared with control. 22

Figure S20. Wikipathways enrichment analysis of proteomic data from 3D hepatocyte spheroids treated with APAP or CAP compared with control. 23

Figure S21. Sankey diagrams of proteomic data from 3D hepatocyte spheroids treated with APAP or CAP compared with control. 24

Figure S22. Schematic diagram of the online mass spectrometry analysis system. 25

Figure S23. Schematic illustration of the automated extraction procedure of the online solid-phase microextraction chip. 26

Figure S24. Mass spectrometry profiles of standard drugs. 27

Figure S25. Standard calibration curves correlating mass spectrometric intensity with concentration for Tamoxifen and 4-OHT. 28

Table 1. The primer sequence used in qPCR assay. 29

·


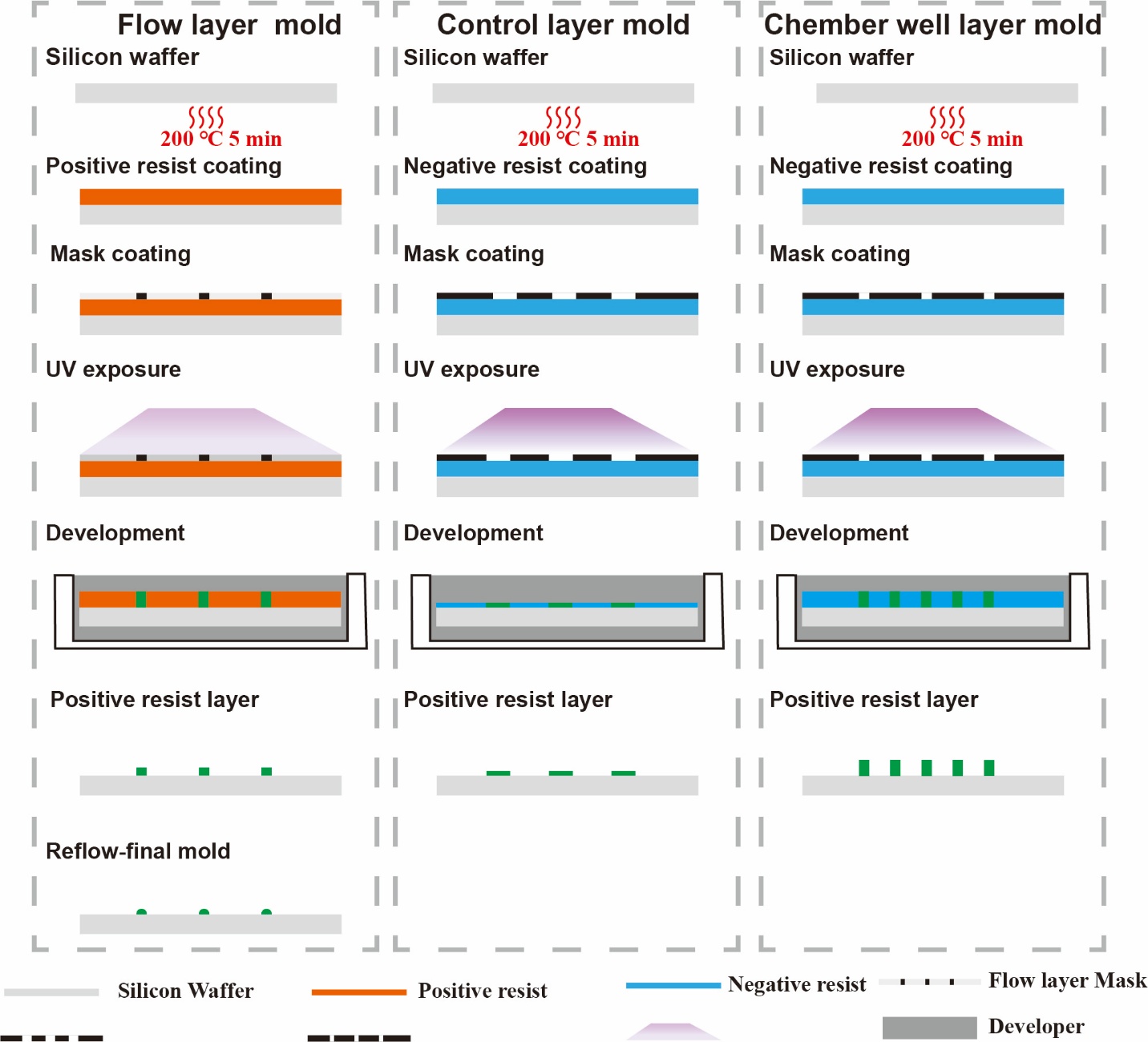


Figure S1. Design and fabrication of the multi-organ microfluidic device. The fabrication of molds was accomplished exclusively by soft lithography. The flow layer mold was patterned using the positive photoresist AZ4620, enabling the formation of rounded arch-shaped channels, whereas the control layer and the microwell array layer were fabricated with the negative SU-8 photoresist.


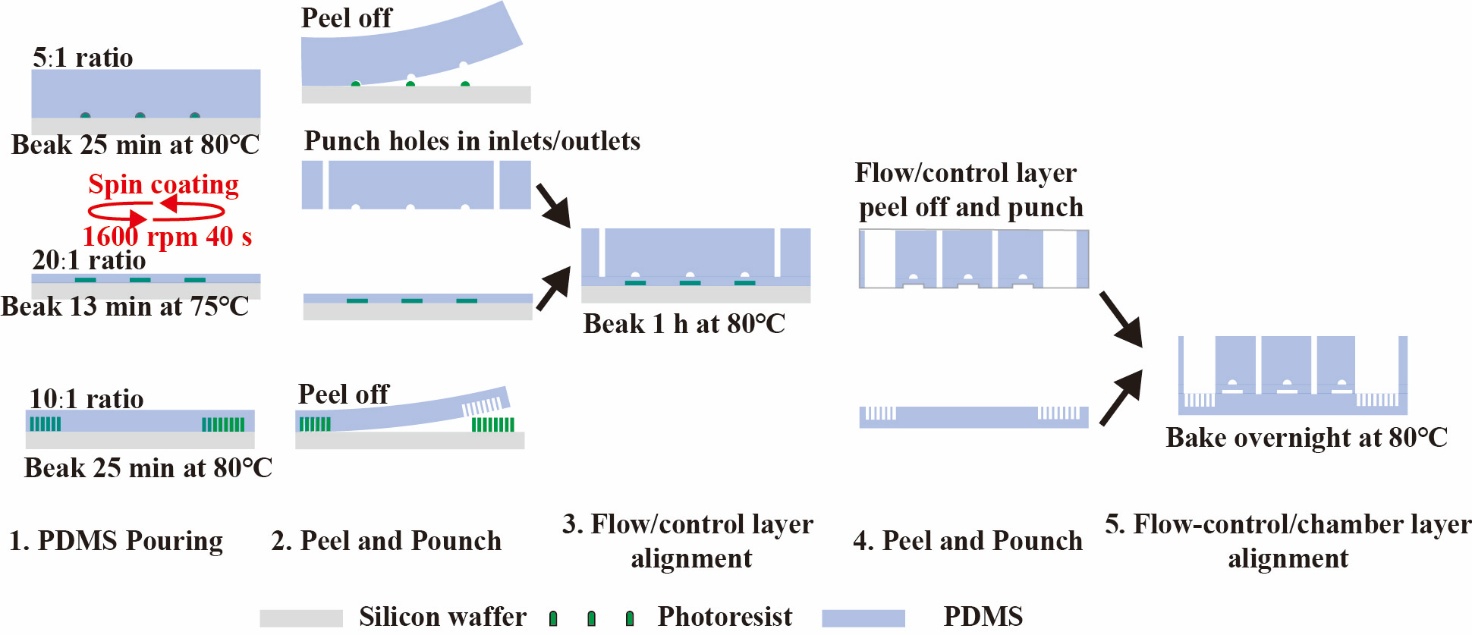


Figure S2. Schematic illustration of the fabrication and assembly of the multi-organ microfluidic PDMS chip. The three PDMS layers were irreversibly bonded together by employing different mixing ratios of PDMS prepolymer and curing agent.


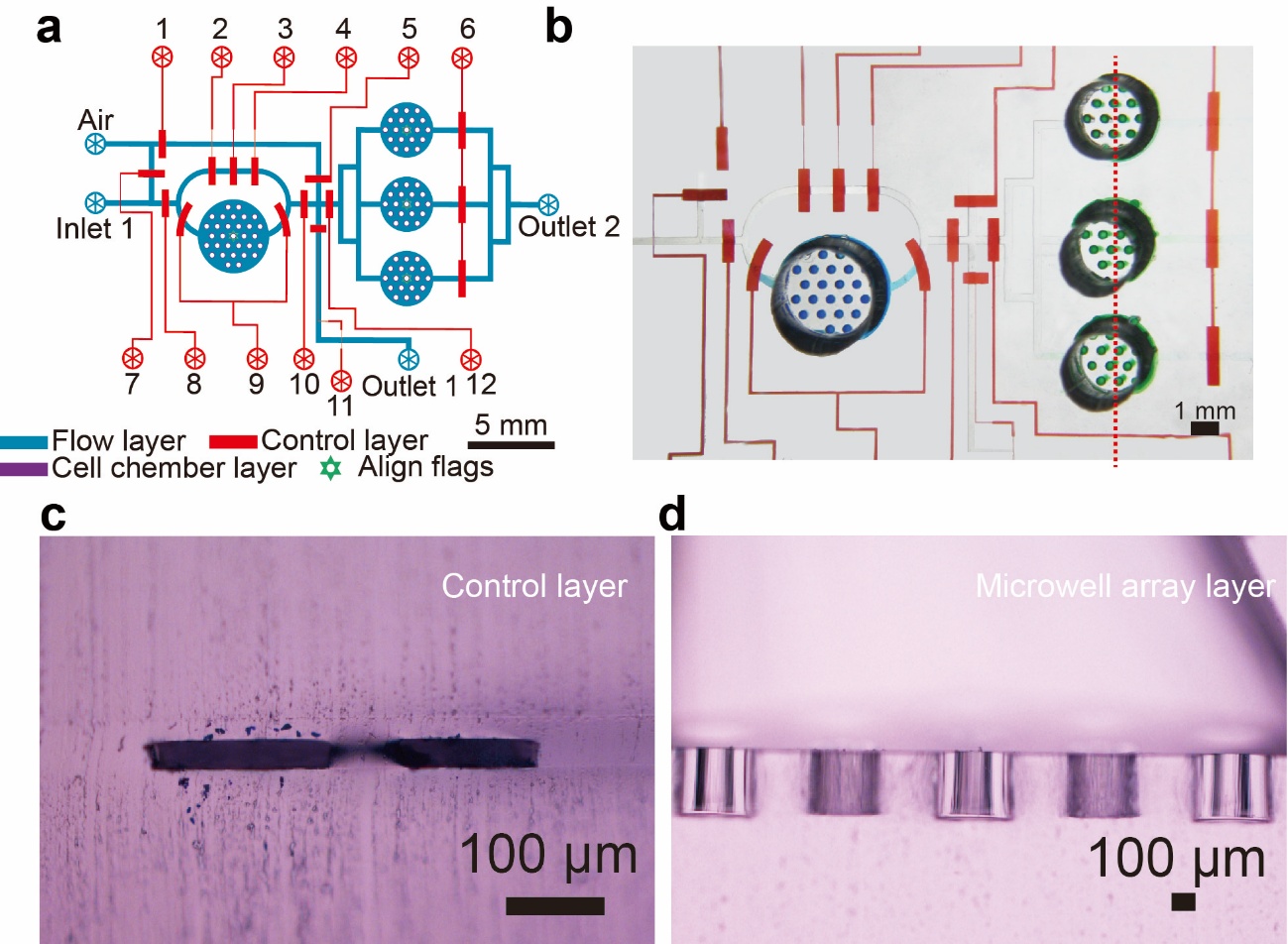


Figure S3. Design and characterization of the multi-organ microfluidic device. (a) CAD design of the chip. (b) Photograph of the fabricated chip. (c) Cross-sectional view of the control layer channel. (d) Cross-sectional view of the array layer of the chip.


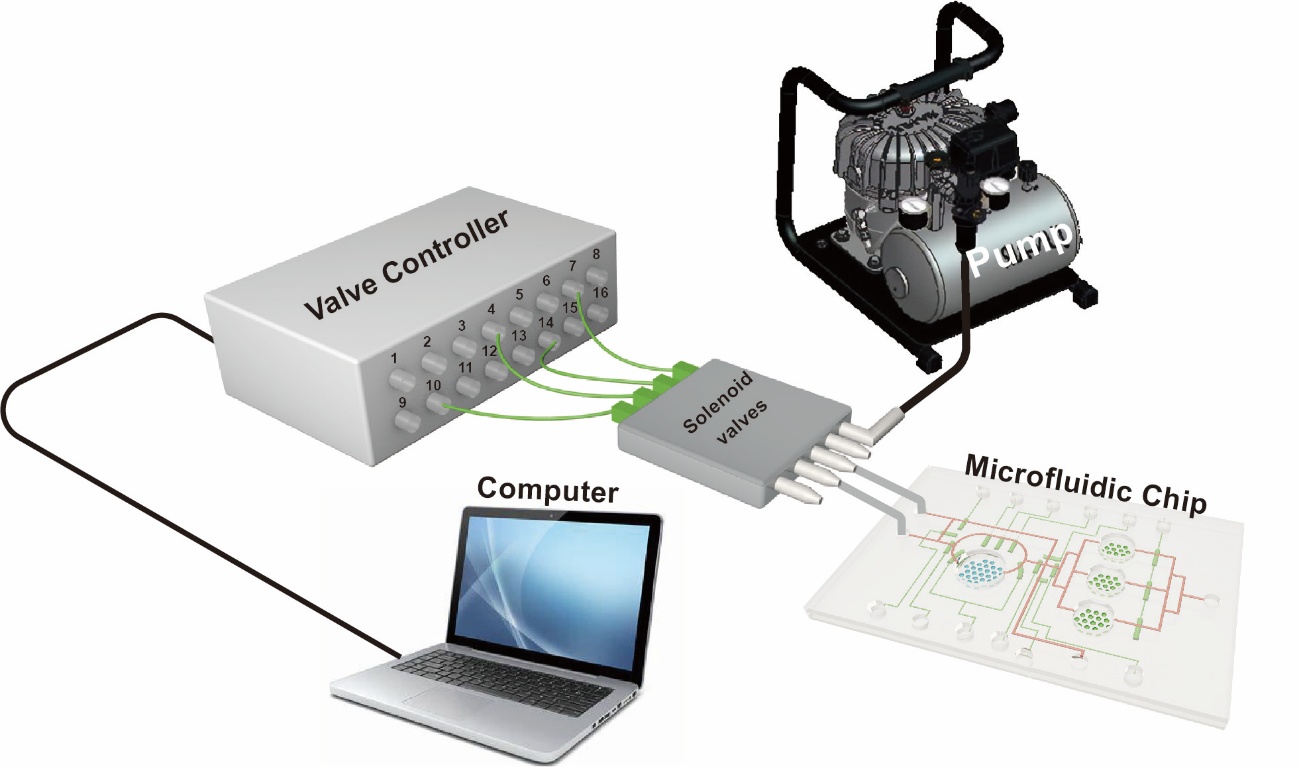


Figure S4. Schematic diagram of an integrated automated microfluidic system. The system consists of four main components: a pump, solenoid valves, a valve controller, and a computer. The pump (top right) serves as the primary driving source, providing the necessary fluid flow to the system. The solenoid valves (center) control the precise flow and distribution of fluids within the microfluidic chip, with their activation managed by the valve controller (top left). The valve controller is connected to the computer (bottom left), which executes the automation process by sending control signals to the solenoid valves. The microfluidic chip (bottom right) is equipped with embedded channels and chambers for fluid manipulation and analysis.


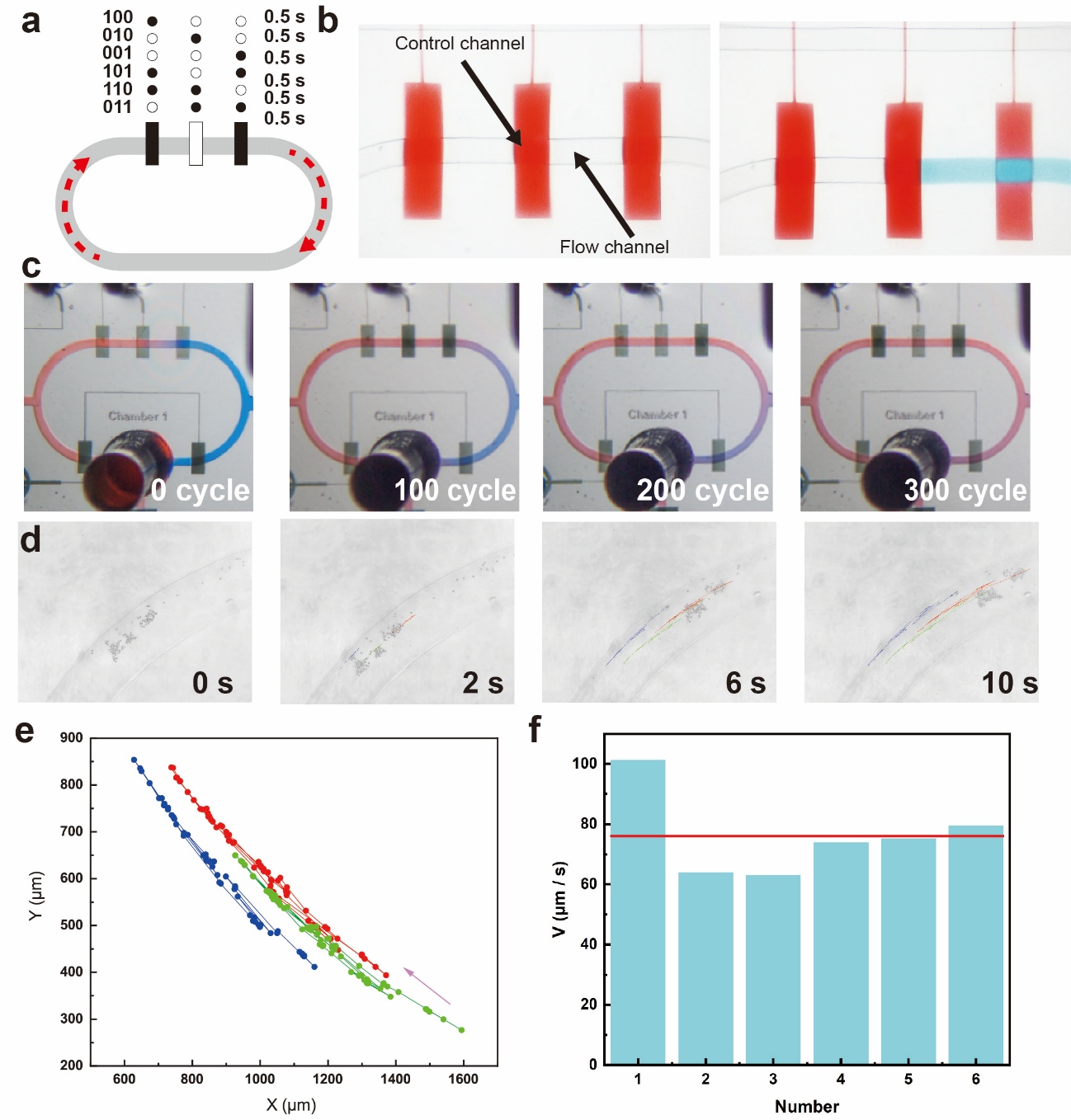


Figure S5. Principle of closed-loop recirculating fluid actuation and flow rate detection. (a) Sequential actuation of the linear array valves. (b) Left: fluid channel not fully filled with solution, with the control valve open; Right: fluid channel fully filled with solution, where the control valve is actuated to block the flow. (c) Dynamic characterization of solution mixing in the closed-loop recirculation region (movie S4). (d–e) Displacement of polystyrene microspheres within the fluid channel over 10 s and the corresponding displacement distribution (movie S5). (f) Statistical analysis of recirculating flow rates within the fluid channel.


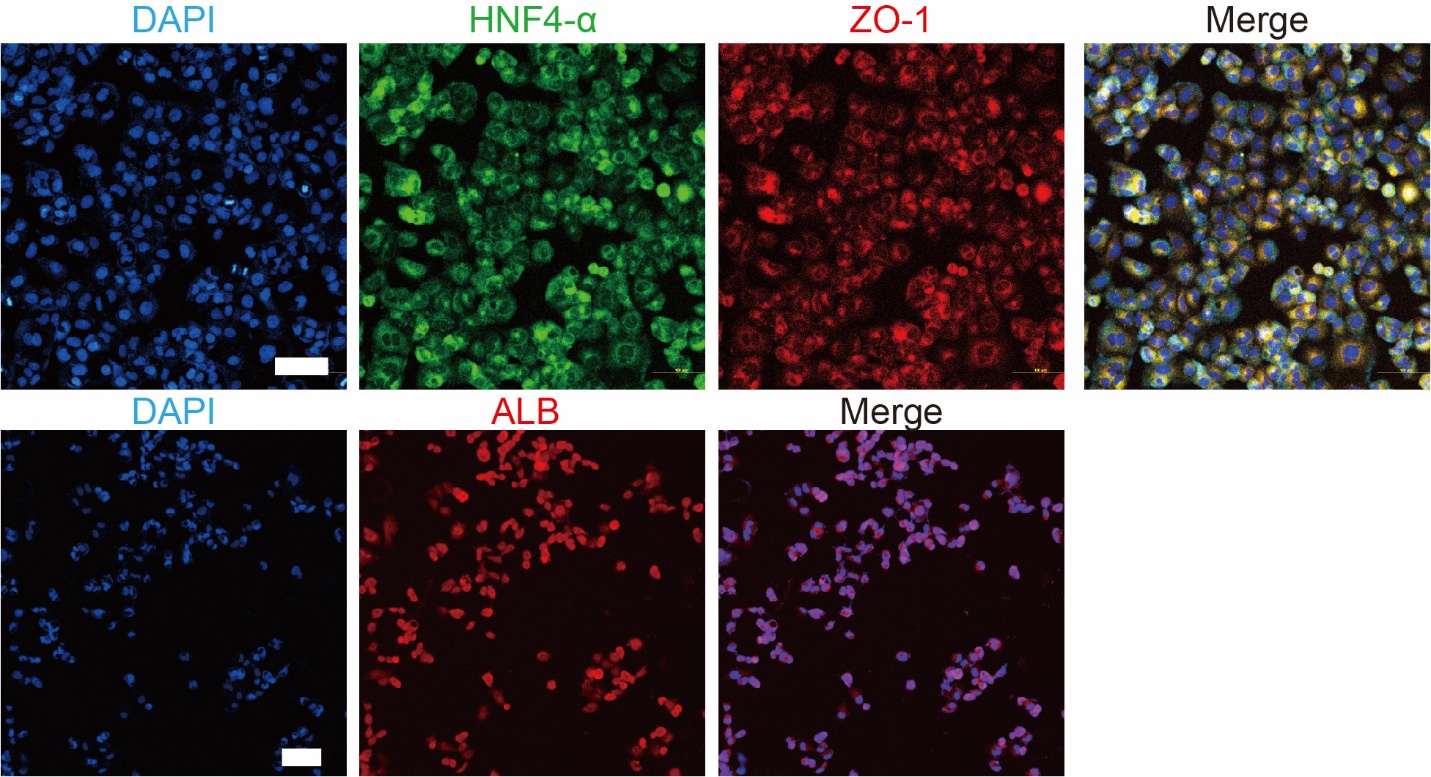


Figure S6. Immunofluorescence micrographs of 2D culture conditions: albumin (ALB, red), hepatocyte nuclear factor 4-alpha (HNF4-α, green), and zonula occludens-1 (ZO-1, red), with nuclear counterstaining (DAPI, blue). Scale bar: 100 μm.

**
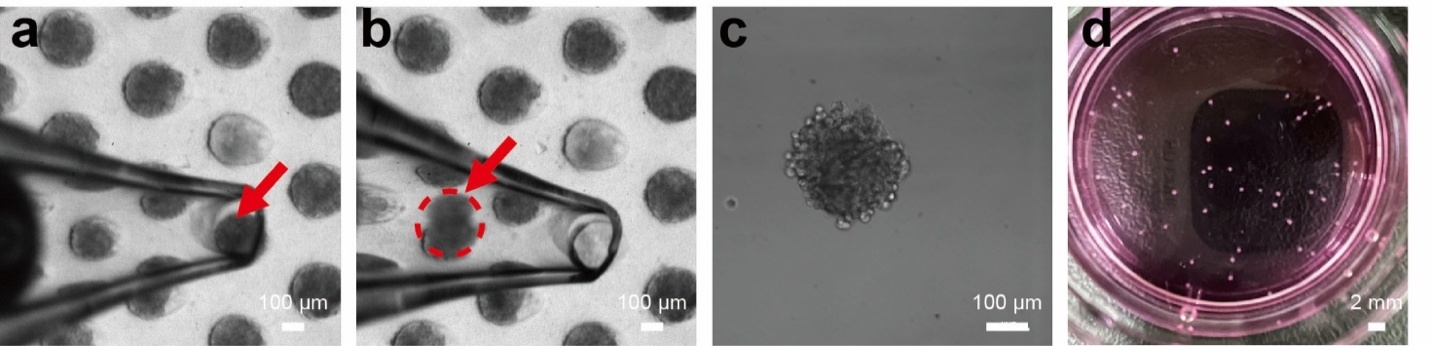
**

Figure S7. Manual selection of 3D liver cell spheroids using a glass pipette on the multi-organ microfluidic chip. (a) The glass pipette (300 µm in diameter) is used to manually pick up a 3D liver spheroid from the micro-well array. (b) The spheroid is positioned within the pipette tip (indicated by the red arrow). (c) The spheroid is aspirated into the pipette and is ready for transfer. (d) The spheroid is deposited into a culture dish, and a brightfield micrograph shows the spheroid after placement on the surface.


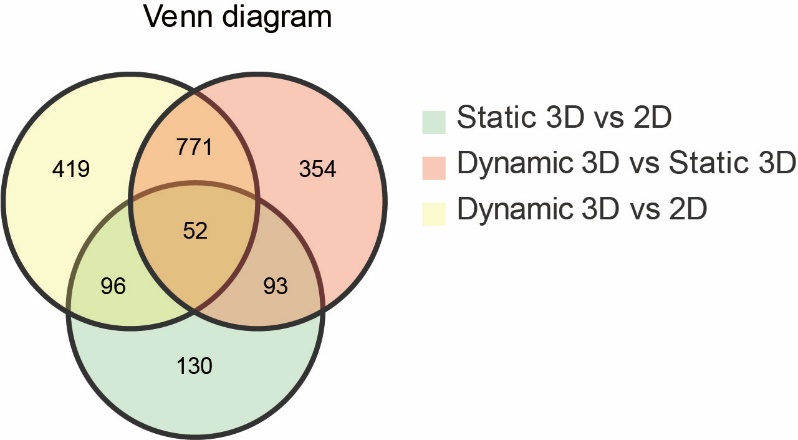


Figure S8. Venn diagram illustrates the overlap of differentially expressed proteins between the culture conditions. The green, red, and yellow regions represent proteins uniquely or commonly regulated in Static 3D vs 2D, Dynamic 3D vs Static 3D, and Dynamic 3D vs 2D comparisons, respectively. The numbers in the intersecting regions indicate the number of shared proteins between the groups.


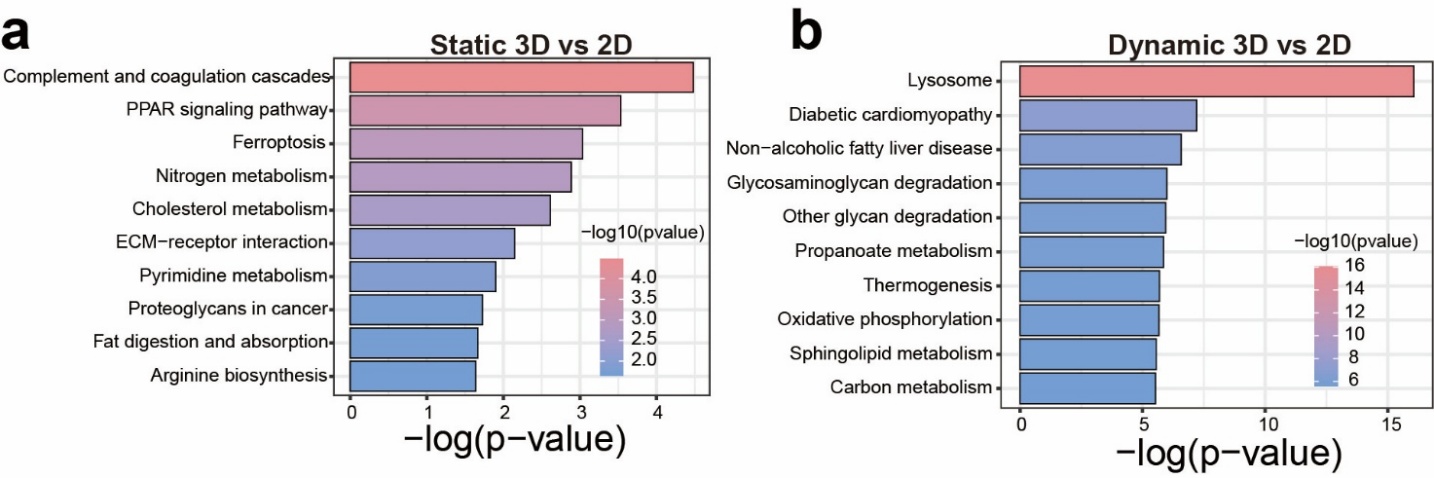


Figure S9. KEGG pathway enrichment analysis of differentially expressed proteins in hepatocyte spheroids cultured under different conditions. (a) Static 3D vs 2D, (b) Dynamic 3D vs 2D. The bar plots display the top enriched KEGG pathways. The x-axis represents the statistical significance (−log₁₀ p-value), and bar color indicates enrichment significance, with red representing higher enrichment. Pathways listed reflect biological processes and signaling cascades significantly influenced by the different culture conditions.


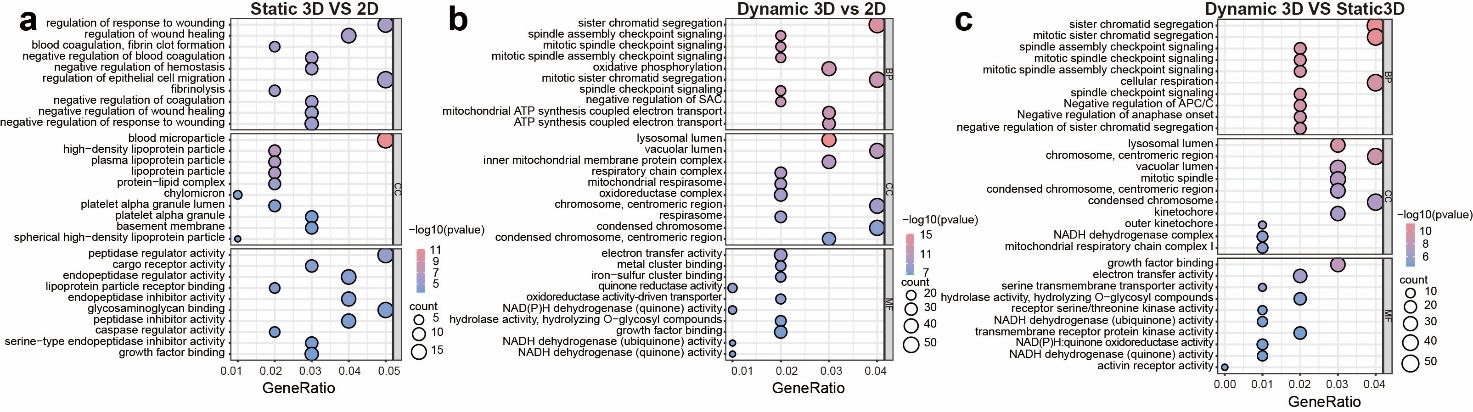


Figure S10. Gene Ontology (GO) enrichment analysis of differentially expressed proteins in hepatocyte spheroids under different culture conditions. (a) Static 3D vs 2D, (b) Dynamic 3D vs 2D, and (c) Dynamic 3D vs Static-3D. Bubble plots display significantly enriched GO terms categorized into biological process (BP), cellular component (CC), and molecular function (MF). The x-axis represents the gene ratio (the proportion of differentially expressed proteins mapped to a specific GO term), bubble size corresponds to the number of proteins, and bubble color indicates statistical significance (−Log₁₀ p-value), with red representing higher significance.


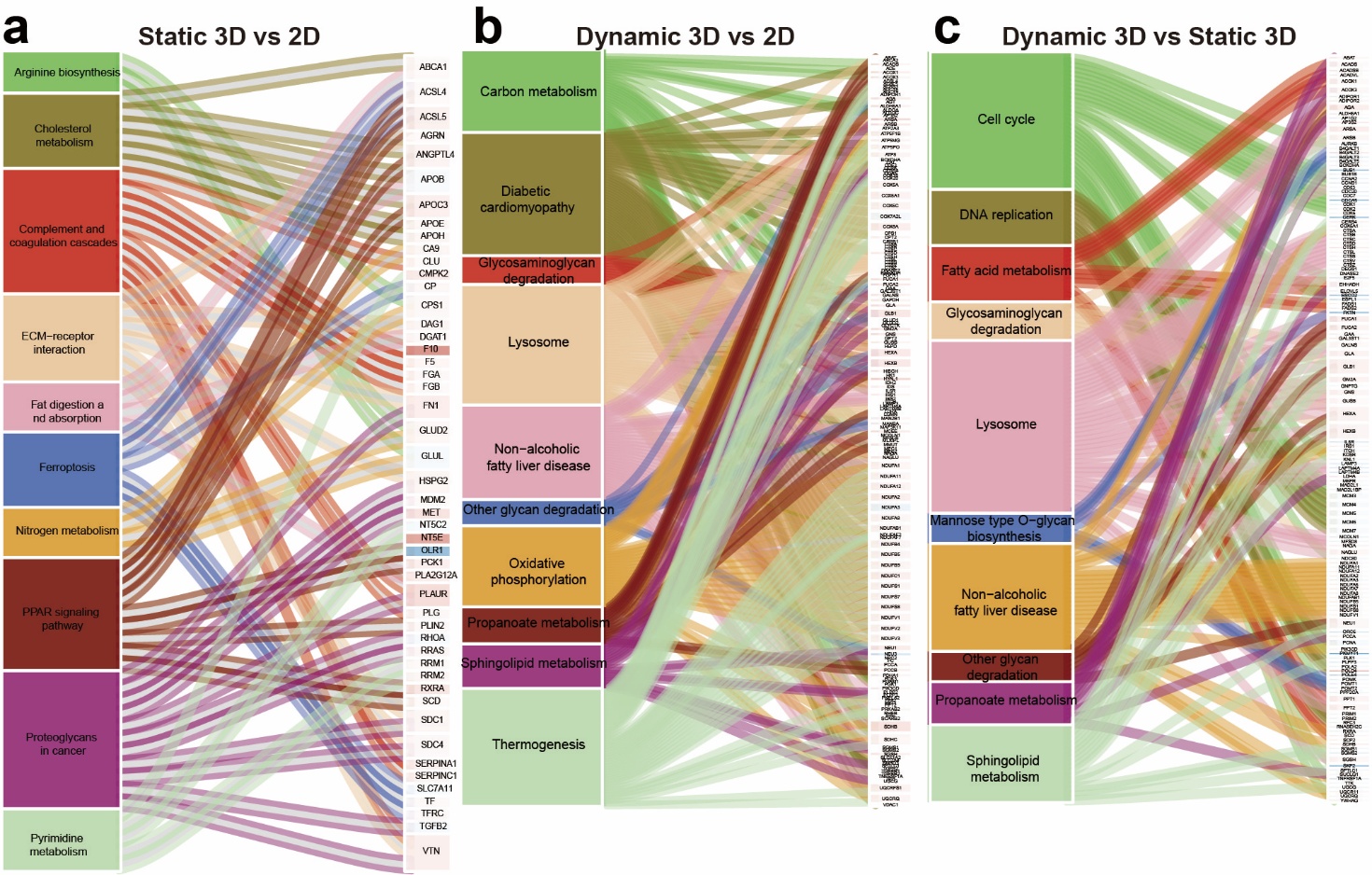


Figure S11. Sankey diagram illustrating differentially expressed proteins in hepatocyte spheroids cultured under different conditions. (a) Static 3D vs 2D, (b) Dynamic 3D vs 2D, and (c) Dynamic 3D vs Static-3D. The diagram links enriched pathways (left) to corresponding differentially expressed proteins (middle) and their interactions (right). The width of the flows represents the number of proteins associated with each pathway, thereby visualizing the complex relationships between culture conditions, molecular functions, and signaling networks.


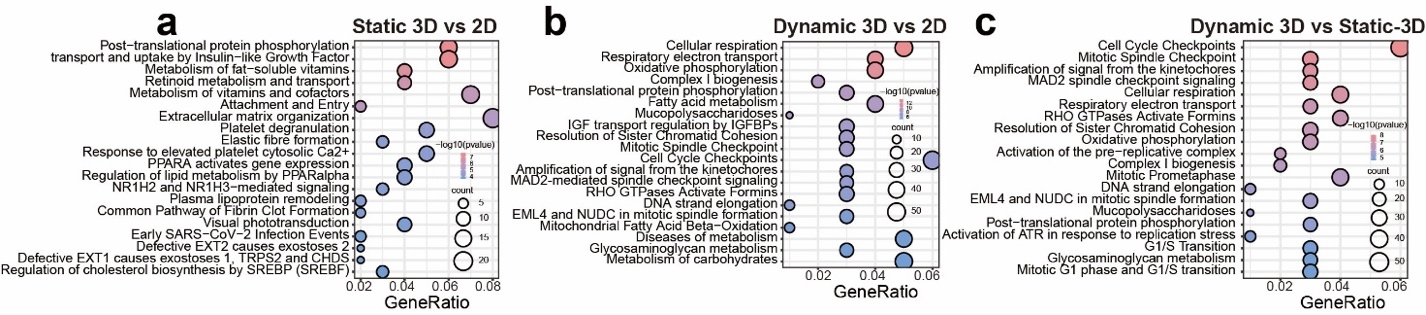


Figure S12. Reactome pathway enrichment analysis of differentially expressed proteins in hepatocyte spheroids under different culture conditions. (a) Static 3D vs 2D, (b) Dynamic 3D vs 2D, and (c) Dynamic 3D vs Static-3D. Bubble plots display significantly enriched Reactome pathways. The x-axis represents the gene ratio (the proportion of differentially expressed proteins mapped to a specific pathway), bubble size corresponds to the number of proteins, and bubble color indicates statistical significance (−Log₁₀ p-value), with red representing higher enrichment.


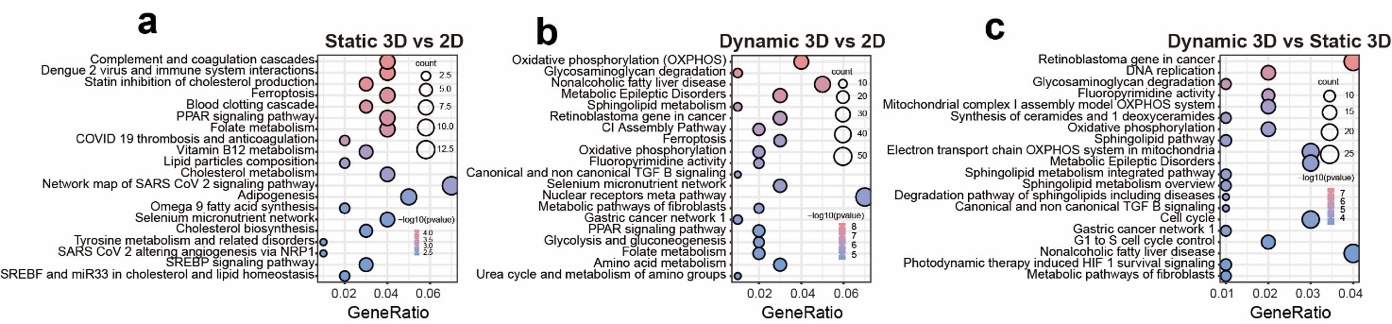


Figure S13. WikiPathways enrichment analysis of differentially expressed proteins in hepatocyte spheroids under different culture conditions. (a) Static 3D vs 2D, (b) Dynamic 3D vs 2D, and (c) Dynamic 3D vs Static-3D. The bubble plots display significantly enriched WikiPathways terms. The x-axis shows the gene ratio (proportion of differentially expressed proteins mapped to each pathway), bubble size reflects the number of associated proteins, and bubble color indicates statistical significance (−Log₁₀ p-value), with red representing higher enrichment.


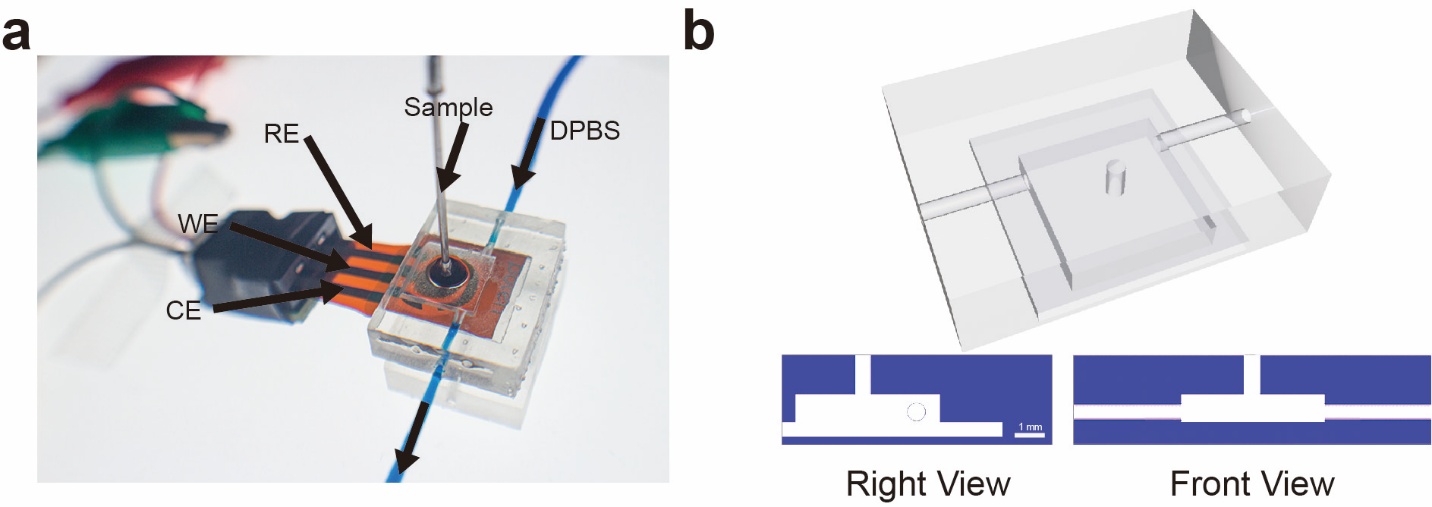


Figure S14. (a) Photograph of the three-electrode system interfaced with the 3D-printed adapter. The working electrode (WE), counter electrode (CE), and reference electrode (RE) are integrated into the adapter. The opposite end of the electrode strip is connected to the electrochemical workstation. (b) Schematic representation of the adapter design, including a 3D perspective view and cross-sectional views (right and front). The chamber structure allows stable electrode–sample contact and efficient fluid exchange, with dimensions on the millimeter scale.


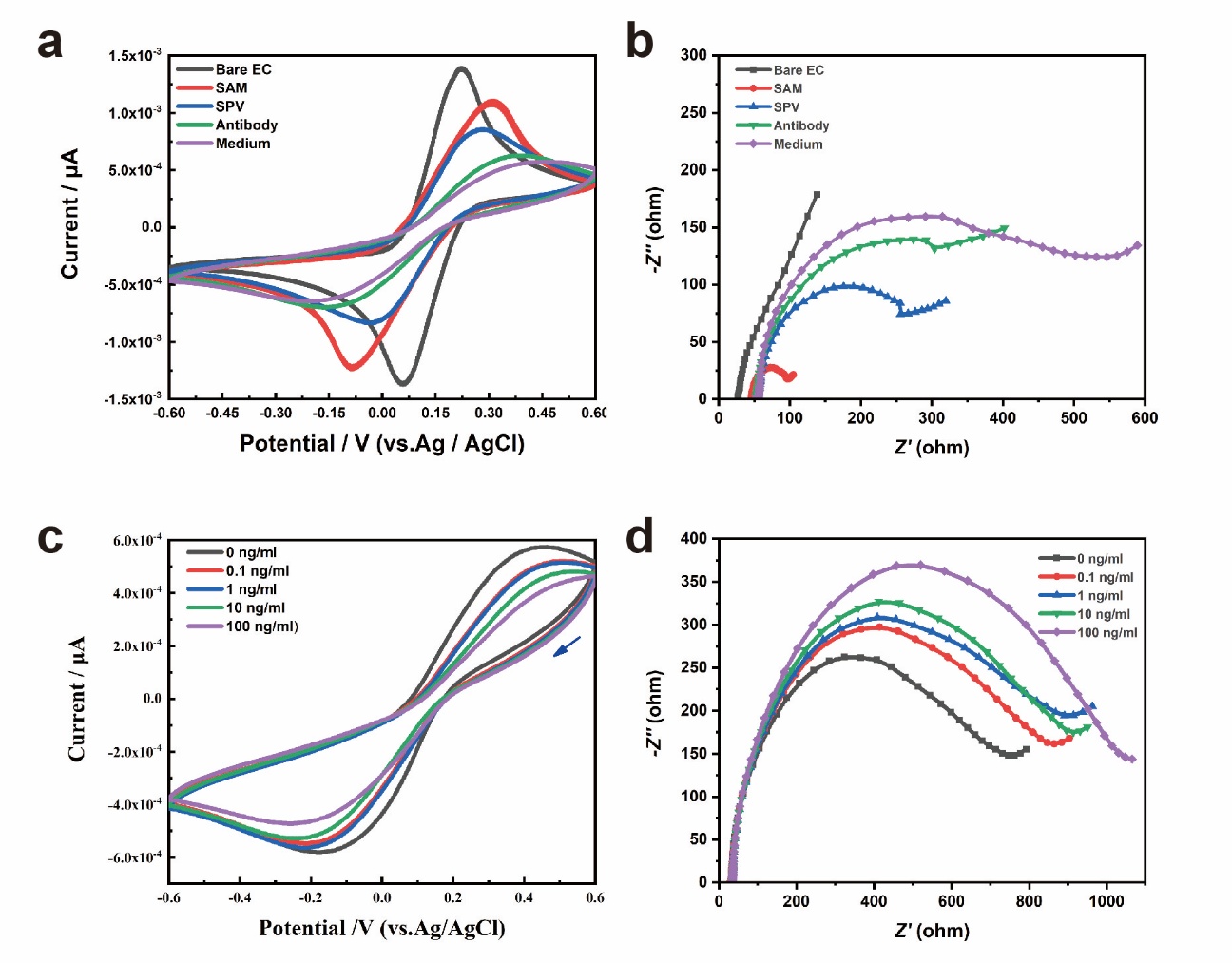


Figure S15. Functionalization of the three-electrode system and detection of albumin standard. (a–b) Cyclic voltammetry and impedance measurements at each step of the electrode functionalization process. (c–d) Cyclic voltammetry and impedance measurements for the detection of albumin standard.


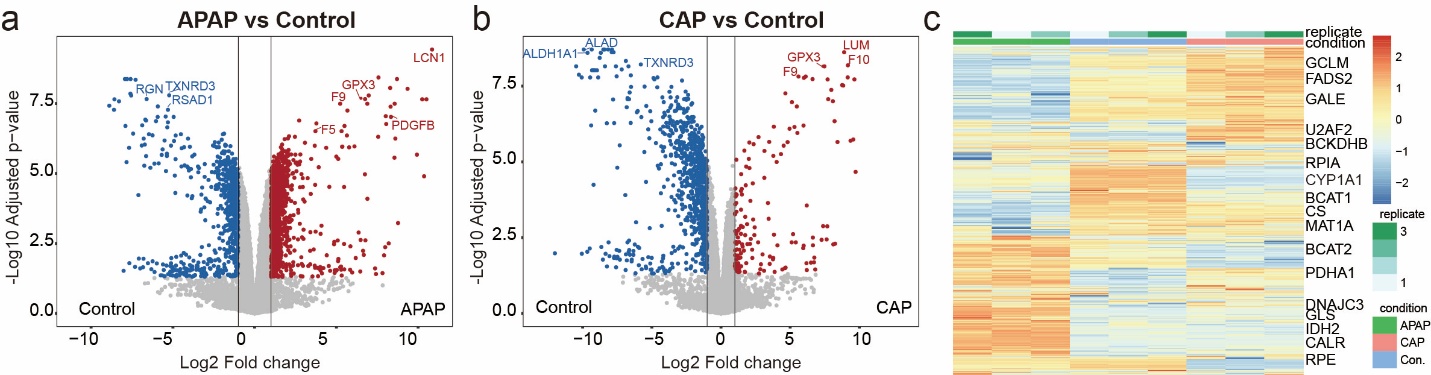


Figure S16. Proteomic profiling of 3D hepatocyte spheroids exposed to APAP, CAP, or control. Volcano plots (left and middle) depict differentially expressed proteins (DEPs) in APAP-treated versus control spheroids and CAP-treated versus control spheroids. Red dots indicate significantly upregulated proteins, blue dots indicate significantly downregulated proteins, and representative proteins are labeled. The heatmap (right) shows hierarchical clustering of DEPs across the three groups, with normalized expression levels color-coded from low (blue) to high (red).


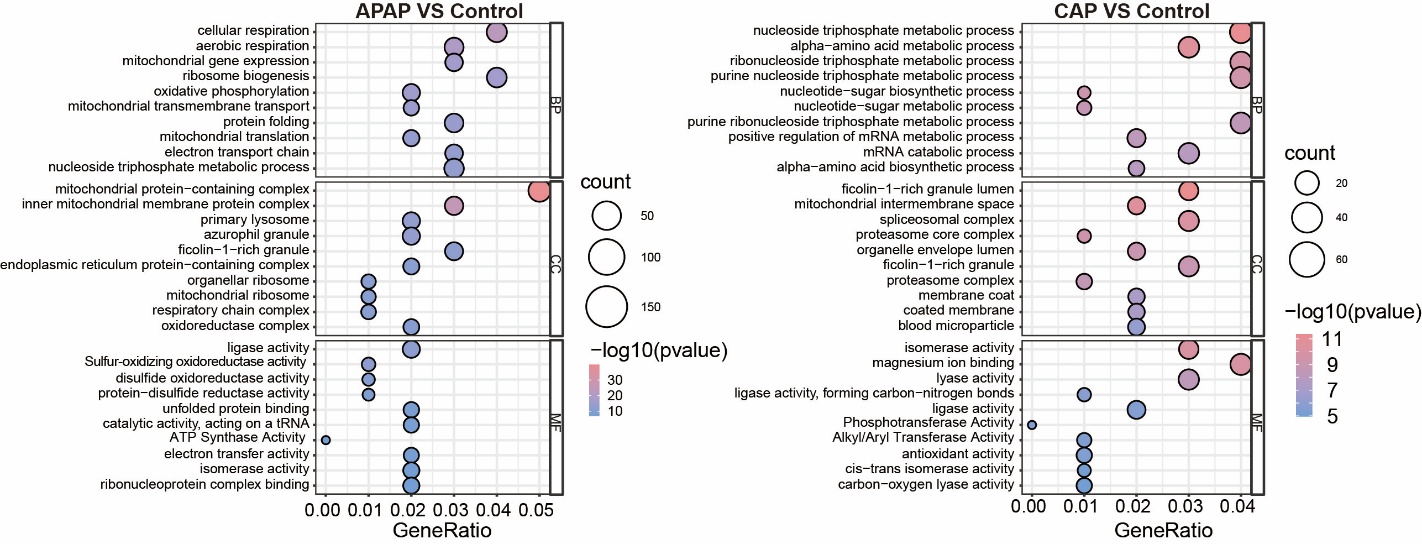


Figure S17. GO enrichment analysis of proteomic data from 3D hepatocyte spheroids exposed to APAP, CAP, or control. Bubble plots show significantly enriched Gene Ontology (GO) terms across biological process (BP), cellular component (CC), and molecular function (MF) categories. The x-axis represents the gene ratio, the bubble size corresponds to the number of enriched proteins, and the color scale indicates statistical significance (–log10 p-value).


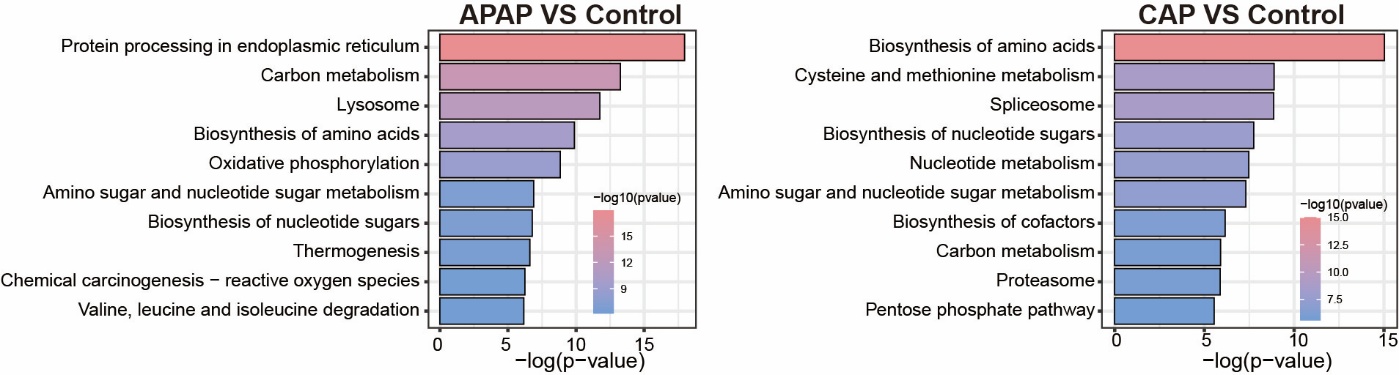


Figure S18. KEGG pathway enrichment analysis of proteomic data from 3D hepatocyte spheroids treated with APAP or CAP compared with control. Bar plots represent the top significantly enriched KEGG pathways in APAP-treated versus control spheroids (left) and CAP-treated versus control spheroids (right). The y-axis lists pathway names, while the x-axis indicates enrichment significance (–log10 p-value). Color gradients denote levels of statistical significance.


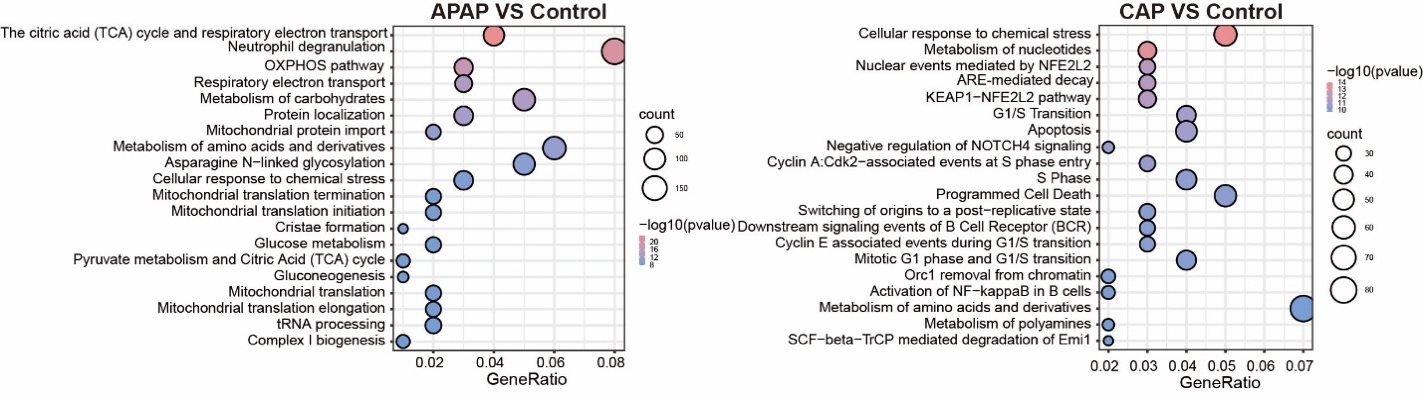


Figure S19. Reactome pathway enrichment analysis of proteomic data from 3D hepatocyte spheroids treated with APAP or CAP compared with control. Bubble plots display significantly enriched Reactome pathways in APAP-treated versus control spheroids (left) and CAP-treated versus control spheroids (right). The x-axis represents the gene ratio, bubble size corresponds to the number of enriched proteins, and color indicates statistical significance (–log10 p-value).


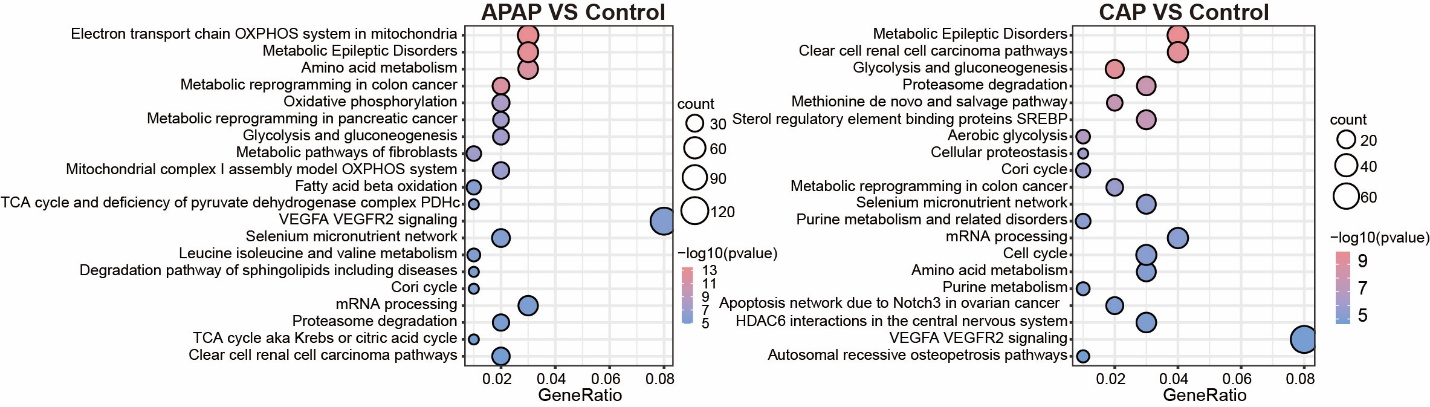


Figure S20. WikiPathways enrichment analysis of proteomic data from 3D hepatocyte spheroids treated with APAP or CAP compared with control. Bubble plots display significantly enriched WikiPathways in APAP-treated versus control spheroids (left) and CAP-treated versus control spheroids (right). The x-axis represents the gene ratio, bubble size corresponds to the number of enriched proteins, and color indicates statistical significance (–log10 p-value).


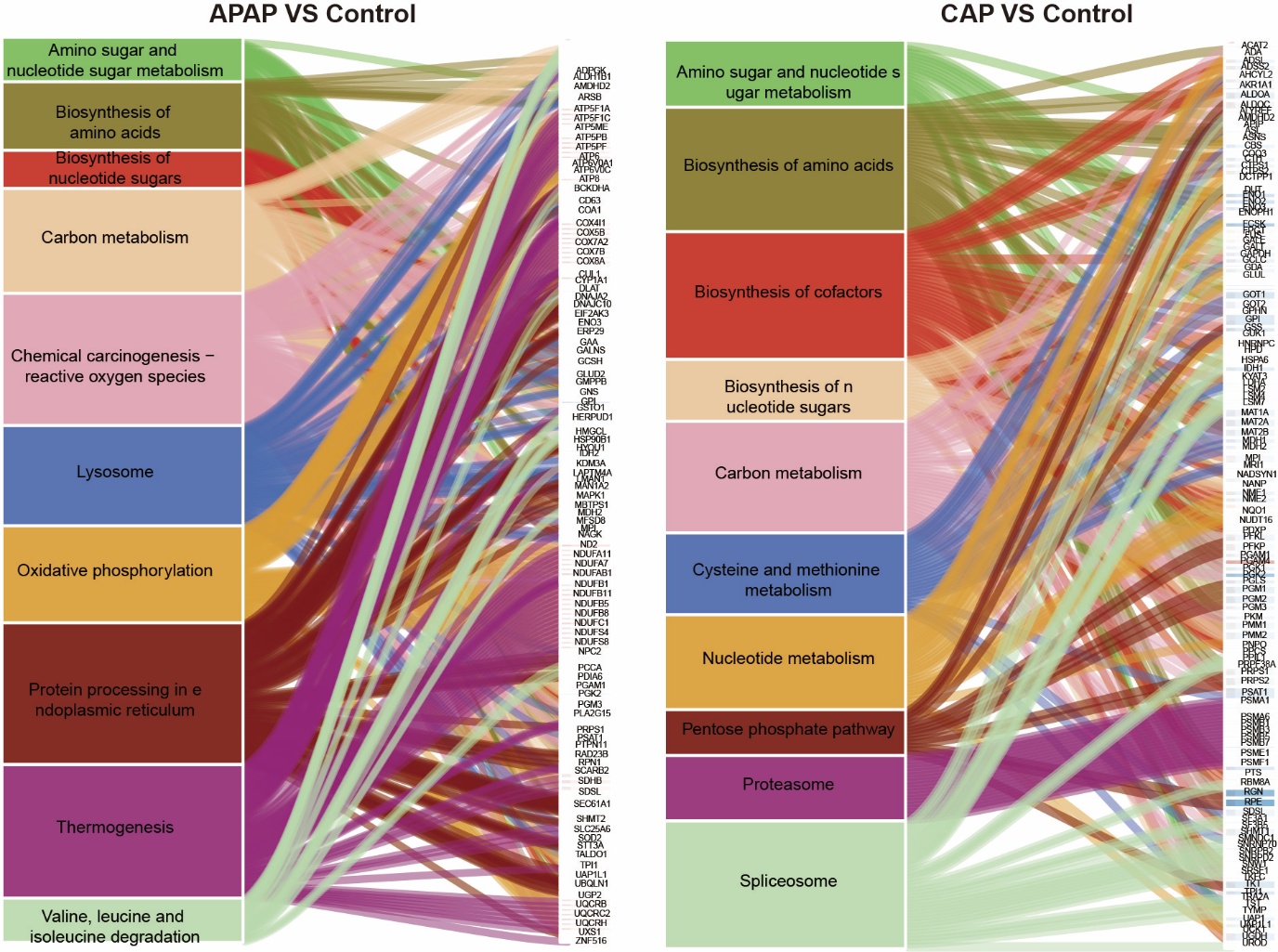


Figure S21. Sankey diagrams of proteomic data from 3D hepatocyte spheroids treated with APAP or CAP compared with control. Sankey diagrams illustrate the relationships between significantly enriched KEGG pathways and their associated proteins in APAP-treated versus control spheroids (left) and CAP-treated versus control spheroids (right). Each pathway is represented by a colored block, with connecting lines indicating the proteins involved in multiple pathways.


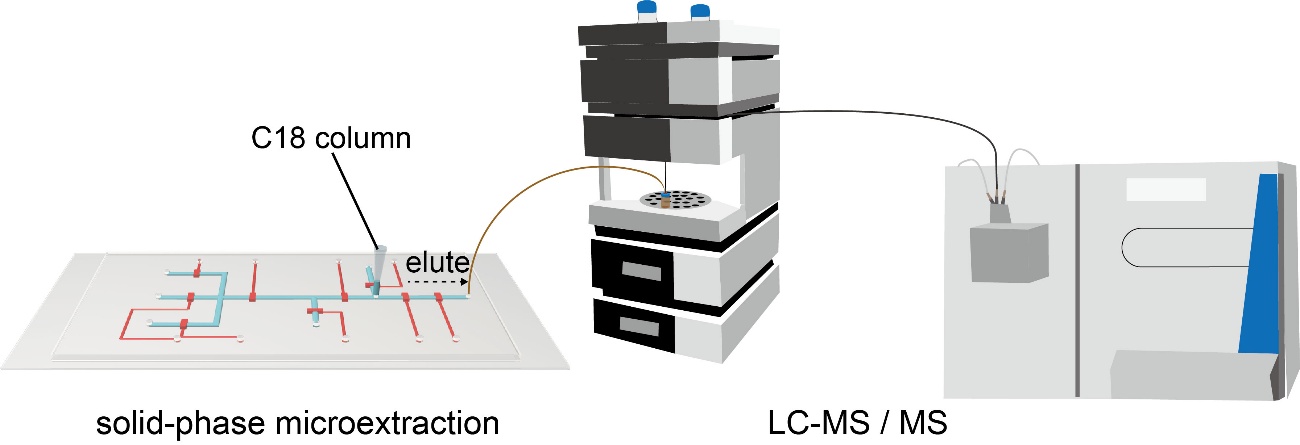


Figure S22. Schematic diagram of the online mass spectrometry analysis system. The automated solid-phase microextraction chip connected to the liquid injection vial via a capillary, with data detected through liquid chromatography-mass spectrometry (LC-MS).


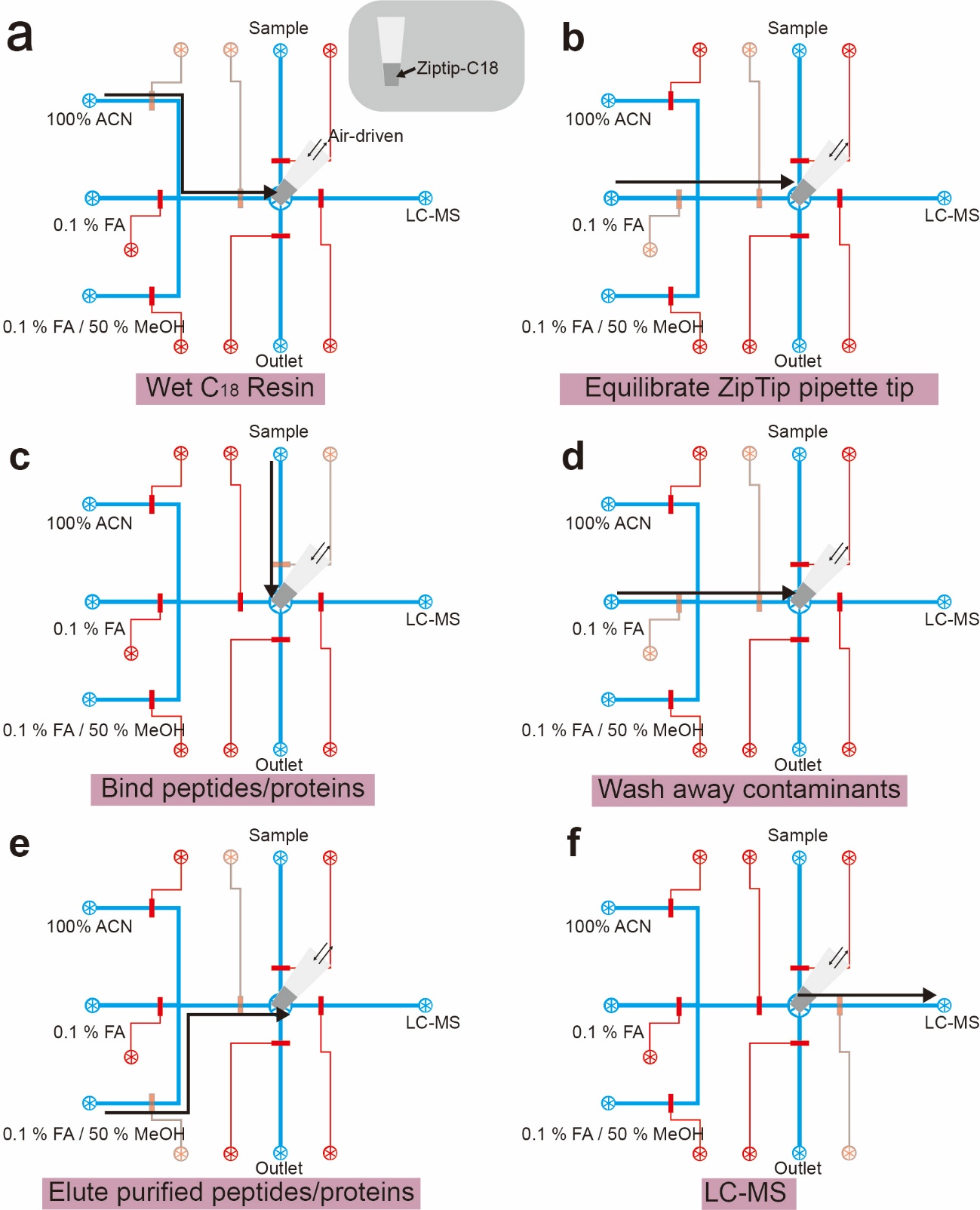


Figure S23. Schematic illustration of the automated extraction procedure of the online solid-phase microextraction (SPME) chip. The SPME process consisted of conditioning, equilibration, loading, washing, and elution. The online extraction was primarily achieved through automated control of the integrated microfluidic chip valves. In the schematic, valves highlighted in light pink indicate the open state, whereas valves shown in dark red represent the closed state.


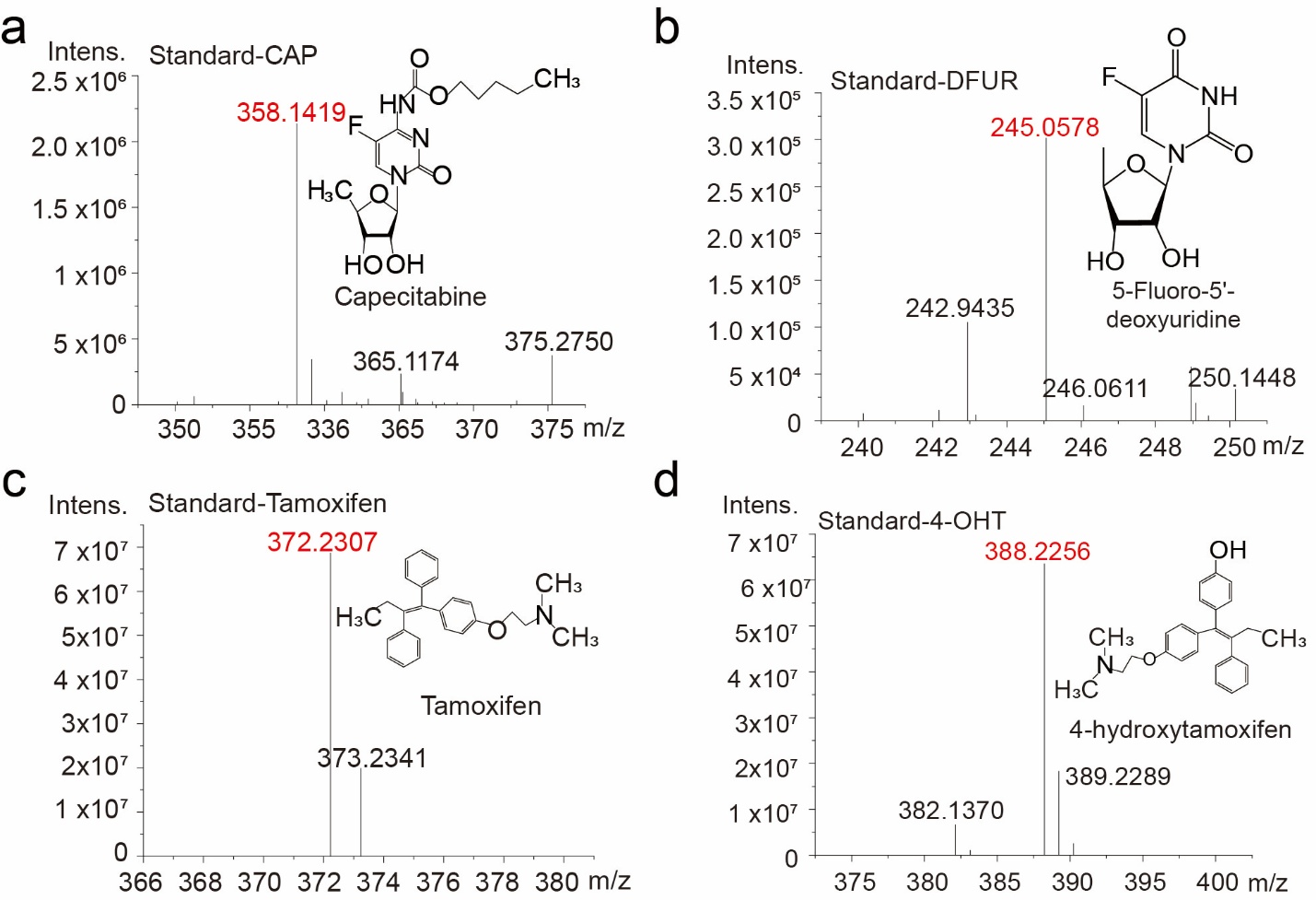


Figure S24. Mass spectrometry profiles of drug standard: (a) Capecitabine, (b) DFUR, (c) Tamoxifen, and (d) 4-OHT, along with their Molecular Structures.


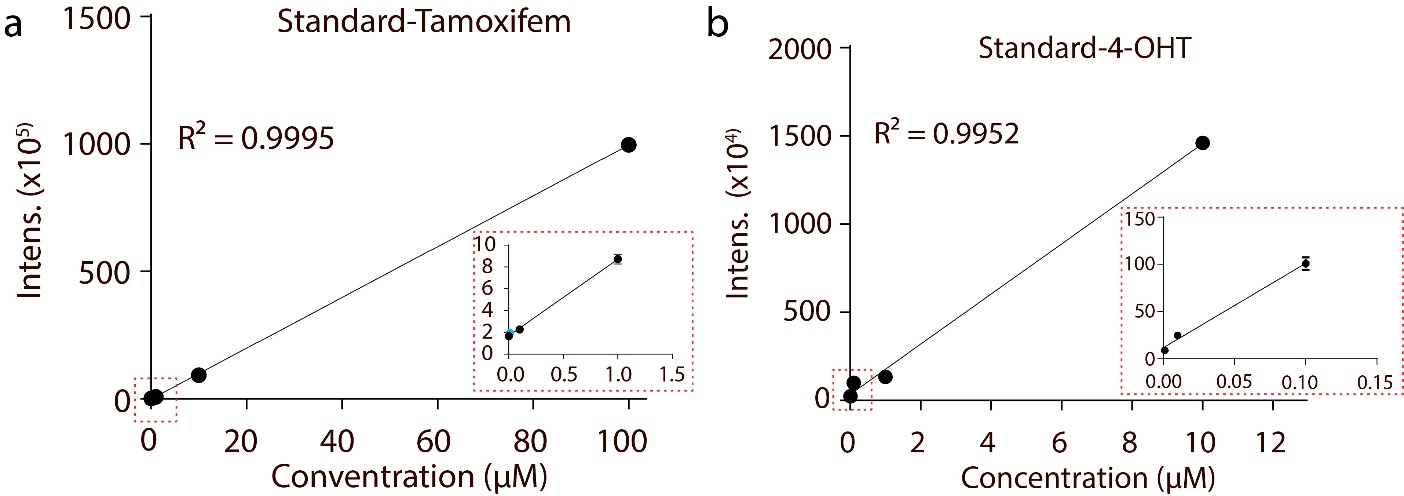


Figure S25. Standard calibration curves correlating mass spectrometric intensity with concentration for (a) tamoxifen (R^2^=0.9995) and (b) 4-OHT (R^2^=0.9952). （The experimental concentrations were as follows: Tamoxifen (0.001, 0.01, 0.1, 1, 10, and 100 μM) and 4-OHT (0.001, 0.01, 0.1, 1, and 10 μM）

| Gene | Forward-primer | Reverse-primer |
| --- | --- | --- |
| CYP2D6 | GAATGCTGTCCCCGTCCT | AGGTCATCCTGTGCTCAGTTAG |
| CYP2C9 | TCCCTGACTTCTGTGCTACATG | ACTGGAGTGGTGTCAAGGTTC |
| CYP2C19 | GAGGAGCATTGAGGACCGT | CACAGCCCAGGATGAAAGT |
| CYP1A1 | AGGGGCGTTGTGTCTTTGTA | CGATAGCACCATCAGGGGTG |
| CFTR | CTGGAATCTGAAGGCAGGAG | GGCATTTCCACCTTCTGTGT |
| ALB | TCGTGCTGCTGCTGAGACT | TTGCTGCCCACTTTTCCT |
| AFP | CTTTGGGCTGCTCGCTATGA | GCATGTTGATTTAACAAGCTGCT |
| β-actin | AAATCTGGCACCACACCTTC | AGAGGCGTACAGGGATAGCA |

Table S1. The primer sequence used in the qPCR assay.

**Supplementary movie (available online)**

**Movie S1:** The Automated inflow of culture medium into the multi-organ co-culture system was evaluated using dye-based assays. (Proof of concept of the Automated inflow of culture medium into the multi-organ co-culture system was evaluated using dye-based assays.)

**Movie S2:** The automated replacement of culture medium in the multi-organ co-culture system was evaluated using dye-based assays. (Proof of concept of the automated replacement of culture medium in the multi-organ co-culture system was evaluated using dye-based assays.)

**Movie S3:** The metabolism and transfer of culture medium in the multi-organ co-culture system using dye-based assays. (Proof of concept of the metabolism and transfer of culture medium in the multi-organ co-culture system using dye-based assays.)

**Movie S4:** Evaluation of mixing efficiency of the chip's embedded peristaltic pump using dye-based method. Proof of concept for peristaltic circulation using food coloring. (A layer of PCR transparent membrane (Kubo Technology, Beijing, China) is sealed on the surface of the chip.)

**Movie S5:** Verification of peristaltic pumping effect and solution flow rate simulation in the chip using 5 µm polystyrene microspheres.

**Movie S6:** Evaluation of the performance of automated solid-phase microextraction using dye-based method. Proof of concept for automated solid-phase microextraction with food coloring.
